# Structure-guided discovery of non-catechol dopamine D1 receptor ligands with biased agonism and antagonism

**DOI:** 10.1016/j.jbc.2026.113076

**Published:** 2026-04-27

**Authors:** Yang Zhou, William C. Wetsel, Alem W. Kahsai, Steven H. Olson, Lawrence S. Barak

**Affiliations:** 1 Department of Cell Biology, Duke University Medical Center, Durham, North Carolina, USA; 2 Departments of Psychiatry and Behavioral Sciences and Neurobiology, Duke University Medical Center, Durham, North Carolina, USA; 3 Department of Medicine, Duke University Medical Center, Durham, North Carolina, USA; 4 Conrad Prebys Center for Chemical Genomics at Sanford Burnham Prebys Medical Discovery Institute, La Jolla, California, USA

**Keywords:** β-arrestin, bias, dopamine D1 receptor, G protein-coupled receptor (GPCR), Parkinson’s disease, structure-based drug design, virtual screening

## Abstract

The catechol L-DOPA, a cornerstone of Parkinson’s disease (PD) treatment, has two major drawbacks: poor pharmacokinetics and, more significantly, debilitating dyskinesias from chronic dopamine D1 receptor (D1R) activation. Preclinical rodent studies suggest that D1R antagonism or β-arrestin-biased agonism can alleviate these motor complications, highlighting the need for next-generation non-catechol ligands. Through virtual screening, we identified eight novel chemotypes as D1R ligands, including two G protein-biased agonists, two β-arrestin-biased agonists and four antagonists. Structure–activity relationship (SAR) optimization led to the development of A82R, a non-catechol D1R antagonist (Ki 733 nM) with high D1 family over D2 family selectivity. Additionally, we present A69, a novel non-catechol β-arrestin-biased partial agonist for D1R (Ki 86.9 nM, stronger than representative D1R commercial drugs) with a sustained half-life of 1 h in the mouse brain. We show that the observed selectivity patterns are consistent with structural and information-theoretic limits on dopamine’s ability to encode receptor subtype identity. Within these bounds, the non-catechol ligand chemotypes represent promising leads for developing therapies that modulate D1R signaling and reduce L-DOPA-induced dyskinesia in PD.

The neurotransmitter dopamine activates a family of 5 G protein-coupled receptors (GPCRs). These receptors are divided into two subfamilies according to their differences in sequence homology and G protein transducers. The first group consists of the dopamine D1 receptor (D1R)—like family composed of the D1R and D5R that are coupled to the Gs protein, and where agonist activation leads to adenylyl cyclase stimulation. The second group, the D2R-like family, consists of the D2R, D3R and D4R that are coupled to Gi/o protein where receptor stimulation inhibits adenylyl cyclase and cAMP production. The D1R and D2R are both highly expressed in key dopaminergic pathways within brain, where they play major roles in regulating locomotion (1), mainly through the direct pathway and the indirect pathway, respectively (2). Such regulatory pathways become abolished in patients with Parkinson’s disease (PD) due to neurodegeneration of dopaminergic projection neurons from the substantia nigra, leading to insufficient dopamine production and sensitization of D1Rs and D2Rs (2). Early PD is treated with D2R family-selective agonists, including pramipexole and ropinirole (3). As the disease progresses, these D2R family-selective drugs become less efficacious and are eventually replaced with the dopamine precursor *L-*DOPA that broadly activates all dopamine receptors (3). However, long-term use of *L*-DOPA can lead to debilitating dyskinesia, primarily through the D1R (4, 5, 6). In this regard, D1R remains a desirable drug discovery target for PD--especially at its late-stage, not only to provide treatment, but also to control dyskinesia. Nevertheless, over the years, an absence of PD drugs expressing profiles with fewer side effects and non-catechol chemotypes has not been forthcoming, and these limitations have impeded the development of new PD therapies (4).

D1Rs activate both G proteins and β-arrestins (βarrs) (4, 7, 8, 9), driving complementary signaling pathways that produce different physiologies. This dichotomy has led to the development of G protein- or βarr-biased compounds that have been found to possess greatly improved side-effect profiles compared to non-biased drugs (10, 11, 12, 13, 14). Overactivation of the G protein pathway downstream of the D1R leads to dyskinesia side effects (5, 15, 16, 17, 18). One way to effectively block D1R G protein activation is through antagonism, which has been shown to prevent dyskinesia at the risk of impairing the effects of *L*-DOPA (19, 20). Additionally, activation of the βarr pathway has been reported to effectively reduce dyskinesia, either through desensitization of the G protein pathway or through its own associated signaling cascade. In preclinical models of PD, βarr2 knockout (KO) mice display pronounced *L*-DOPA-induced dyskinesia through G protein-dependent signaling, whereas viral-induced overexpression of βarr in these KO mice reduced the *L*-DOPA dyskinesia side effects (13, 16, 17, 18, 20). Besides its role in dyskinesia, βarr signaling may also independently regulate locomotion, as suggested in the studies where deletion of βarr2 impeded the beneficial effects of *L*-DOPA-stimulated locomotion (13).

Historically, the catechol moiety has served as the foundation for the development of almost all FDA approved and investigational anti-parkinsonian drugs; these include *L*-DOPA, apomorphine, A-86929, SKF-81297, and dihydrexidine. While these drugs represent a mainstay for PD therapy, the catechol ligands suffer from both poor oral bioavailability and CNS penetration (4). In a high-throughput screening (HTS) campaign, in 2014 Pfizer introduced a first-in-class series of orthosteric D1R non-catechol drugs that were strongly G_s_ protein-biased relative to βarr-biased (7). However, Pfizer’s three D1R G-biased lead candidates for PD, PF-06412562, PF-06649751 (tavapadon), and PF-06669571 failed to meet efficacy endpoints in several Phase IB and Phase II studies (4). Of these compounds, only PF-06649751 remains under consideration in late-stage clinical trials (21).

Attempts to use the PF-06 scaffold and conventional medicinal chemistry approaches to generate D1R compounds have not identified βarr-biased ligands (22, 23). This suggests a link between the chemical scaffold and the failure to produce signaling bias. In this regard, next generation novel D1R chemotypes for antagonists and βarr-biased agonists remain an important challenge for PD drug discovery.

We believe that alternative strategies are required to produce novel therapeutic D1R ligands for PD. Virtual screening, in which drugs are processed in the discovery stage by software algorithms rather than by a first pass through an *in vitro* system, is a relatively new cost- and time-effective methodology that has been used to identify novel GPCR chemotypes (24). Successful virtual studies currently include the identification of novel biased ligands binding to the orthosteric pocket of the D4R (24, 25), as well as the identification of βarr-biased allosteric modulators at D1R (26). In the present study, we assessed one million compounds by virtual screening at the orthosteric pocket of the D1R. With this approach, we have successfully identified druggable PD leads with functional bias. We also evaluated their selectivity across the dopamine receptor family, given the highly conserved orthosteric pockets and the long-recognized lack of subtype selectivity among PD drugs such as L-DOPA, apomorphine, and rotigotine (27).

## Results

### D1R structure evaluation, model selection, and validation

**Figure 1:**
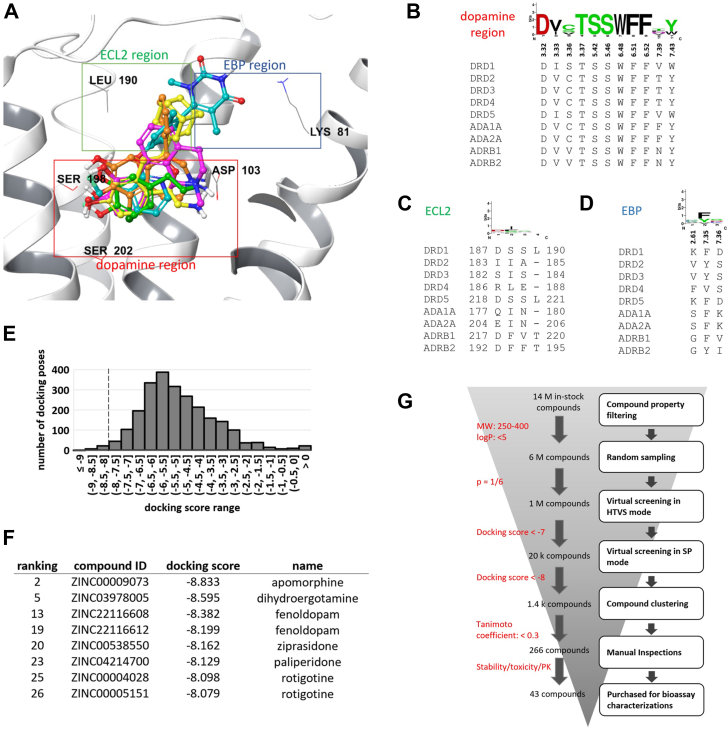
**D1R structure evaluation and virtual-screening model-validation.***A*, subregions within the orthosteric binding pocket of the human D1R (hD1R) and superpositions of different ligand poses (*green*: dopamine; *pink*: apomorphine; *orange*: SKF-81297; *yellow*: A-77636; *cyan*: PW0464). *B–D*, sequence alignments of dopamine and adrenergic receptors at the orthosteric region (*B*), the ECL2 region (*C*), and the region of the extended binding pocket (EBP) (*D*). *E*, histogram of docking scores for FDA-approved small molecule drugs docked to the hD1R structure 7JVQ. The proposed docking score cutoff of −8 is labelled with a vertical dashed line. *F*, table of docking poses that have scores superior to −8 and belong to known D1R ligands. *G*, flowchart illustrating the virtual screening decision paradigm for identifying compounds binding to the orthosteric D1R pocket.

Based on several cryo-EM structural images of the orthosteric ligand-bound human D1R (hD1R), we parsed the hD1R orthosteric binding pocket into three subregions available for ligand occupancy. Region 1 was defined as dopamine-accessible, region 2 contained extracellular loop 2 (ECL2), and region 3 formed an extended binding pocket (EBP) (Fig. 1*A*). Region 1, the deep orthosteric dopamine-binding pocket, is shown in Figure 1*A* as a red box. It contains residue D103 located on transmembrane domain (TM) 3 that forms an ionic interaction with the positively charged amine group on catecholamine and D1R drugs. Similarly, residues S198 and S202 on TM5 form hydrogen bonds with the ligand’s hydroxyl groups, and a group of aromatic residues on TM7 forms π–π stacking interactions with the catechol benzene. Sequence alignment between dopamine and adrenergic receptors reveals that the region in the binding pocket is highly conserved, explaining binding promiscuity among catecholamines with these residues (Fig. 1*B*).

The ECL2 region contains space proximate to hD1R ECL2. It is accessible to D1R selective ligands like SKF-81297 and A-77636 (Fig. 1*A*, green box). With the exception of the D5R, the alignments of dopamine and adrenergic receptors with the hD1R’s ECL2 reveal this region is not conserved between the D1R and these other receptors (Fig. 1*C*). Additionally, the hD1R family ECL2 is a single amino acid longer than the hD2R family ECL2 indicating why SKF-81297 and A-77636 are D1R family selective. The EBP region is defined by the space proximate to TM2, and by example, it is accessible to the non-catechol ligand PW0464 (Fig. 1*A*, cyan). The alignments of the dopamine and adrenergic receptor sequences relative to their EBP regions indicate the D1R family contain positively charged lysine residues in this region *versus* the aliphatic or aromatic residues found on the other receptors (Fig. 1*D*). Together, these structural evaluations provide a foundation for achieving dopamine receptor selectivity.

We chose the D1R apomorphine-bound structure 7JVQ as our screening target following the superposition and analysis of six cryo-EM structures with different bound orthosteric ligands (Fig. S1). Most residues in the orthosteric pockets overlapped well, with a few variations noted at D103, S202 and N292. Considering that some structures bound agonist and others bind antagonists, we observed that the orthosteric pocket underwent only minor structural changes with ligand type. To assess the general performance of 7JVQ as a model for virtual screening, we docked structures derived from 1614 FDA-approved small-molecule drugs retrieved from the ZINC database. They resulted in 2819 unique enantiomers and possible tautomers at physiological pH. Docking the 2819 structures under the software standard precision-mode produced 2425 docking poses. The docking scores follow a right-skewed near-normal distribution with a mean at −5.09 and a standard deviation of 1.55 (Fig. 1*E*). Twenty-nine of the 2425 poses (1.2%) had docking scores superior to −8.00. Among them were eight docking poses belonging to six known D1R drugs. Also identified in the study were a diverse set of drugs, including the catechol ligands apomorphine and fenoldopam, the ergot alkaloid dihydroergotamine, the non-ergoline drug rotigotine, and the atypical antipsychotics ziprasidone and paliperidone that are known also to bind at the hD1R (28, 29) (Fig. 1*F*).

### Virtual screening at the orthosteric pocket of D1R

Using the 7JVQ structure, we performed virtual screening targeting the hD1R orthosteric pocket (Fig. 1*G*). We first applied filters of molecular weights between 250 and 400, and with a logP less than 5 to 14 million compounds in the ZINC database that were commercially available and in stock. This approach resulted in a library of 6 million compounds, from which 1 million were randomly selected and processed using the software’s high-throughput virtual screening (HTVS) mode. The top 2% of candidate compounds with a docking score cutoff of −7.00 were further screened in standard precision mode to give comparable docking scores with validation of the models. This screen identified 1439 hits with docking scores superior to −8.00. Compound clustering to identify similar chemical structures was performed using a Tanimoto coefficient cutoff of 0.3. This grouping identified 266 unique chemotypes. These computer calculations on a single core using an Intel i7-3770k CPU took a total of 577505 s or roughly 6 days and 19 h.

Upon further validation, we discovered these virtual screening hits occupied the hD1R in the dopamine-binding orthosteric region with hydrogen-bonding interactions at TM5 and π-π stacking interactions at TM6. We also determined that some hits occupied the ECL2 region, displaying hydrogen-bonding interactions at Ser188 and with hydrophobic interactions at L190. Some hits interacted with K81 through ionic interactions, hydrogen bonds, or cation–π interactions. After assessing the chemical stabilities and potential toxicities associated with these chemotypes, we purchased 43 compounds (Fig. 1*G*).

### Discovery of novel chemotypes with biased agonists and antagonists at D1R

**Figure 2:**
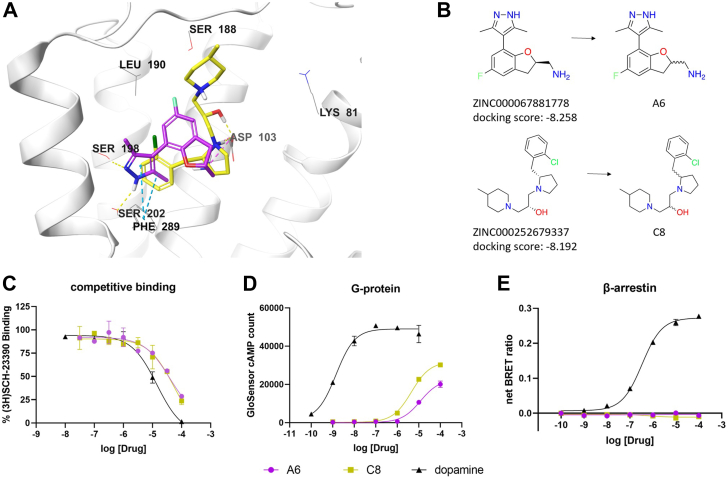
**Discovery of novel G protein-biased partial agonists at the D1R.***A*, predicted docking poses of ZINC67881778 (*purple*) and ZINC252679337 (*yellow*) bound to the orthosteric pocket of the hD1R. *B*, Structures of the virtual screening hits. *C–E*, dose-dependent responses in competition binding assays (*C*) of A6 (*purple*), C8 (*yellow*). And dopamine (*black*) at the D1R; (*D*) GloSensor cAMP; and (*E*) BRET βarr2 recruitment assays. N = 3 independent experiments. Error bars represent ± SD.

We used competitive radioligand binding with [^3^H]-SCH23390 on membranes from transfected D1R cells to evaluate the 43 virtual hits. At 100 μM, eight of these displaced more than 50% of the [^3^H]-SCH23390 from the D1R. Additional D1R functional assays characterized the hits as 2 G protein-biased agonists, 2 βarr-biased agonists, and 4 antagonists. The 2 G protein-biased agonists, A6 and C8, have chemotypes distinct from currently known D1R orthosteric ligands and each other (Fig. 2*B*). They occupy the dopamine-binding region of the orthosteric pocket, displaying hydrophobic interactions with TM7 and ionic interactions with Asp103 (Fig. 2*A*). Both agonists have mid-range micromolar affinities (Fig. 2*C*) and possess low micromolar potencies for cAMP accumulation (Fig. 2*D*); but without any βarr activity (Fig. 2*E*). Without hD1R transfected, A6 and C8 did not induce cAMP accumulation, confirming that ligand induced cAMP accumulation was mediated by hD1R (Fig. S2*A*). Under a confocal microscope, these ligands showed no βarr-mediated translocation, and they blocked dopamine-induced receptor translocation (Fig. S2*B*). Together, these results suggested that A6 and C8 are novel G protein-biased D1R partial agonists.

**Figure 3:**
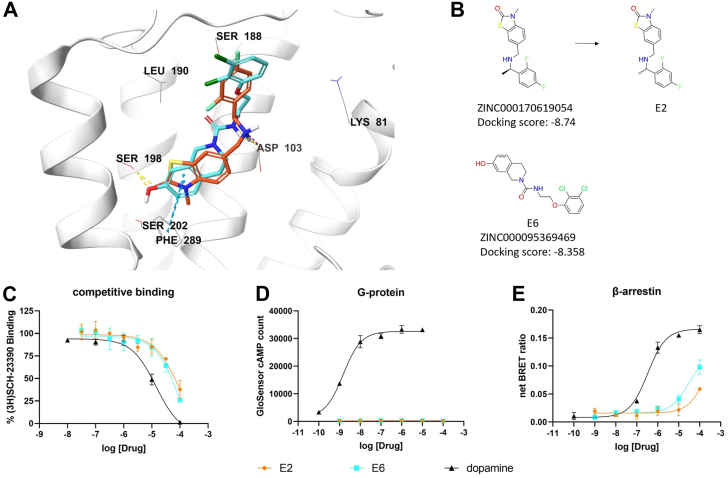
**Discovery of novel β-arrestin-biased partial agonists at the hD1R.***A*, Predicted docking poses of ZINC170619054 (*orange*) and ZINC95369469 (*cyan*) bound to the orthosteric pocket of the hD1R. *B*, structures of the virtual screening hits and the stereochemically undetermined compounds E2 and E6 that were purchased and tested. *C–E*, dose-dependent responses of E2 (*orange*), E6 (*cyan*), and dopamine (*black*) at the hD1R in (*C*) competition binding, (*D*) GloSensor cAMP, and (*E*) BRET βarr2 recruitment assays. N = 3 independent experiments. Error bars represent ± SD.

We identified compounds E2 and E6 as novel βarr-biased partial agonists. They have distinct chemotypes (Fig. 3*B*). Nevertheless, it is striking that they form C-shaped binding modes, occupy the same space within the hD1R orthosteric binding-pocket, and engage the receptor with similar types of chemical interactions. Both compounds form hydrogen bonds with Ser198 on TM5, contain aromatic rings predicted to form π-π stacking interactions with Phe289 on TM6, and their amine groups form either hydrogen bonds or ionic interactions with Asp103 on TM3. Additionally, each ligand has an aromatic ring in the ECL2 region of the hD1R, with halogen substitutions pointing towards Leu190 on ECL2 (Fig. 3*A*). They bind the orthosteric site with mid-range micromolar potencies (Fig. 3*C*), possess no activity in the G protein signaling pathway (Fig. 3*D*) but demonstrate βarr-mediated activity (Fig. 3*E*). At 100 μM concentration, both compounds produced robust βarr-mediated receptor translocation (Fig. S3). Together, these results indicate E2 and E6 are novel βarr-biased partial agonists.

**Figure 4:**
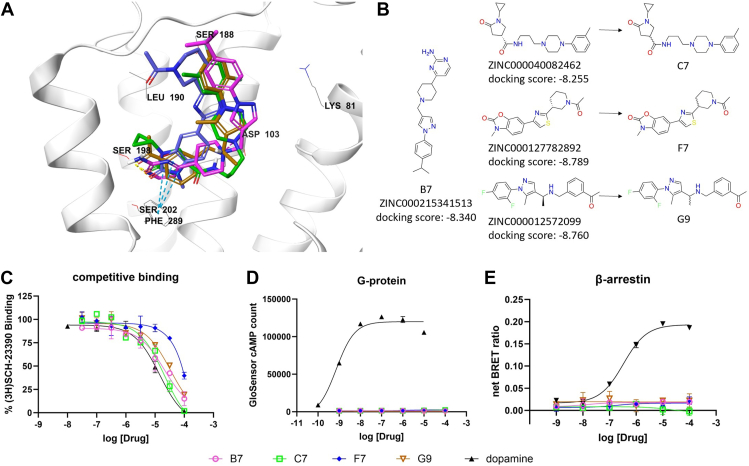
**Discovery of novel antagonist of the hD1R.***A*, Predicted docking poses of ZINC215341513 (*pink*), ZINC40082462 (*green*), ZINC127782892 (*blue*), and ZINC12572099 (*brown*) bound to the orthosteric pocket of the hD1R. *B*, structures of the virtual screening hits for the stereochemically non-used compounds B7, C7, F7 and G9 that were purchased and tested. *C–E*, dose-dependent responses of B7 (*pink*), C7 (*green*), F7 (*blue*), G9 (*brown*), and dopamine (*black*) at the hD1R in (*C*) competition binding assay, (*D*) GloSensor cAMP assay, and (*E*) BRET βarr2 recruitment assay. N = 3 independent experiments. Error bars represent ± SD.

We identified compounds B7, C7, F7, and G9 as novel hD1R orthosteric antagonists (Fig. 4*B*). Each possesses a unique chemotype distinct from previously known orthosteric ligands. Structural modeling revealed these compounds, like the βarr-biased agonists, adopt a C-shaped conformation enabling them to interact with the hD1R in a similar manner (Fig. 4*A*). All four compounds form hydrogen bonds with Ser198 on TM5 through acceptor atoms. Additionally, B7, F7, and G9, but not C7, engage in π-π stacking interactions with Phe289 on TM6. B7, C7, and G9--but not F7--form ionic interactions with Asp103 on TM3 due to their positively charged amine groups. Finally, each compound extends an additional ring structure into the ECL2 region.

Competitive radioligand binding confirmed these compounds bind the hD1R orthosteric site with midrange micromolar potencies (Fig. 4*C*). None activated G protein signaling (Fig. 4*D*) and, likewise, none recruited βarr (Fig. 4*E*), suggesting a complete absence of receptor activation. Importantly, each compound effectively blocked dopamine-induced cAMP accumulation (Fig. S4*A*) and receptor translocation (Fig. S4*B*), confirming their antagonist action.

### Structure–activity relationship optimization of antagonist G9

**Figure 5:**
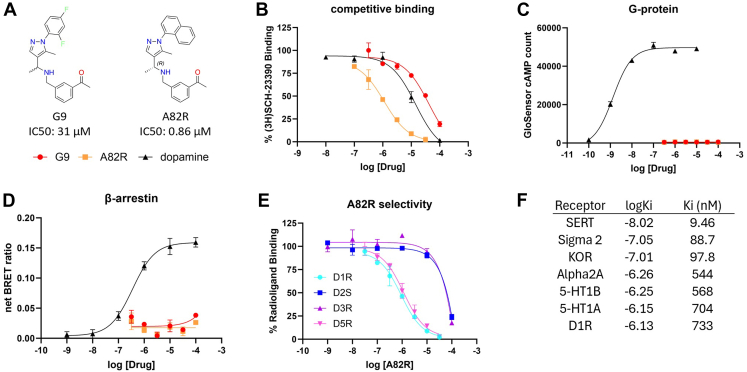
**Structure-activity relationship (SAR) optimization of the rigid C-shaped antagonist G9.***A*, structures of the C-shaped antagonist G9 and its optimized lead A82R discovered through SAR optimizations. *B–D*, dose-dependent responses of G9 (*red*) and A82R (*orange*) at the hD1R in (*B*) competition binding, (*C*) GloSensor cAMP, and (*D*) BRET βarr2 recruitment assays. *E*, competition binding of A82R at hD1R (*cyan*), hD2S (*blue*), hD3R (*purple*), and hD5R (*magenta*). *F*, PDSP GPCRome radioligand binding screening of A82R against a panel of GPCR targets in the brain. Targets with Ki less than 1 μM are listed, with full table in Table S1 and individual dose response curves in Fig. S6. N = 3 independent experiments. Error bars represent ± SD.

To develop a lead compound in the novel non-catechol antagonist category, we optimized the antagonist hit G9. Our medicinal chemistry effort yielded A82R as a lead compound (Fig. 5, *A* and *B*). It is a submicromolar hD1R antagonist, with neither G protein nor βarr activity (Fig. 5, *C* and *D*). Consistent with our hypothesis that engaging the ECL2 region may lead to receptor selectivity, A82R demonstrated strong selectivity to hD1R over the D2R receptor family (Fig. 5*E*). A82R was further submitted to the NIMH Psychoactive Drug Screening Program (PDSP) (30) to be screened against 41 drug targets in the brain, which identified seven targets with Ki values less than 1 μM, with Ki value at D1R determined to be 733 nM and a surprisingly strong affinity of 9.46 nM at the serotonin transporter (SERT) (Figs. 5*F*, S5, and Table S1).

### Structure activity relationship optimizations of the β-arrestin-biased hit E2

**Figure 6:**
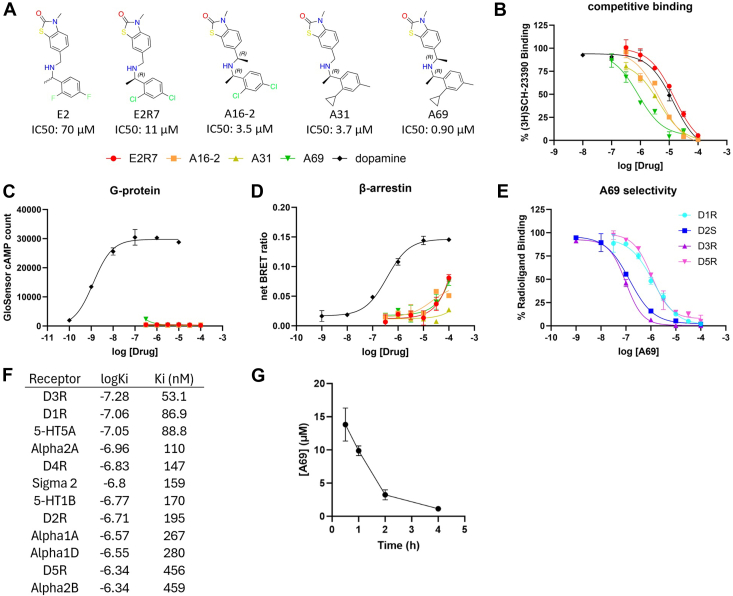
**Structure-activity relationship (SAR) optimization of the βarr-biased hit E2.***A*, structures of the βarr-biased hit E2 and its milestone analogs E2R7, A16-2, A31, and A69 discovered through SAR optimizations. *B–D*, dose-dependent responses of E2R7 (*red*), A16-2 (*orange*), A31 (*yellow*), A69 (*green*), and dopamine (*black*) at the hD1R in (*B*) competitive binding, (*C*) GloSensor cAMP, and (*D*) BRET βarr2 recruitment assays. *E*, competition binding of A69 at hD1R (*cyan*), hD2S (*blue*), hD3R (*purple*), and hD5R (*magenta*). *F*, PDSP GPCRome radioligand binding screening of A69 against a panel of receptor targets in the brain. Targets with Ki less than 500 nM are listed, with full table in Table S2 and individual dose response curves in Fig. S7. *G*, pharmacokinetic analysis of brain drug concentrations in mice detected after 30 mg/kg i.p. administration. N = 3 independent experiments. Error bars represent ± SD.

To develop a lead compound in the novel non-catechol β-arrestin-biased agonist category, we performed extensive structure-activity relationship studies around the βarr-biased hit E2 to improve its hD1R affinity by synthesizing and testing over 100 analogs. E2 has a chiral center (Fig. 6*A*) and, consistent with our modeling prediction, the R stereoisomer had a higher affinity for the hD1R than the other compound. The R stereoisomer E2R7, with both fluorine atoms substituted by chlorine produced a compound that was an additional 6-fold more potent than E2 and after introduction of an extra carbon and an extra chiral center, A16-2, provided another 3-fold improvement. In parallel, using E2R7, we generated the 3-fold more potent A31 by substituting the two chlorines with an ortho cyclopropane group and a *para*-methyl group. The result of combining these modifications into a single compound was A69 (IC_50_: 0.90 ± 0.18 μM). The structures and competition displacement curves for these intermediates and A-69 are shown in Figure 6, *A* and *B*. These E2 analogs contained no G protein activity (Fig. 6*C*), but they demonstrated βarr2 activity (Fig. 6*D*).

As A82R demonstrated strong selectivity to D1 family receptors over D2 family receptors, we postulated that E2 and its analogs would also be D1R/D5R selective due to their interaction with the relatively distinct amino acid motif expressed by ECL2. To our surprise, A69 affinity measurements at the different dopamine receptor subtypes revealed it bound most strongly to hD2R short and hD3R (Fig. 6*E*), with an hD3R affinity (IC_50_: 76 ± 20 nM) around 13-fold higher than the hD1R affinity. A69 was shown to be an antagonist at the hD3R (Fig. S6, *A* and *B*). Confirmatory docking of A69 to hD1R and hD2R produced docking scores of −8.745 and −9.024, respectively. Inspection of the docking pose at hD1R showed A69 maintained the C-shaped binding mode with the cyclopropane group pointing towards ECL2 (Fig. 6C, left). At the hD2R, the ECL2 region was smaller and more constraining to the ligand, leaving no space for the cyclopropane group (Fig. 6D, left). Due to the flexibility of the A69 compound at the hD2R, especially at the four bonds connecting the two aromatic rings, the benzene ring can adjust its orientation to place the cyclopropane ring into the EBP region (Fig. 6D, right), which is notably larger in hD2R than hD1R (Fig. 6C, right).

We also submitted A69 to PDSP for screening analysis against a broader panel of receptors. This analysis revealed that A69 has a D1R Ki value of 86.9 nM and confirmed stronger D3R affinity with Ki value of 53.1 nM (Fig. S7). Additionally, out of the 42 brain receptors tested by PDSP, the 12 receptors with the highest affinity included all five dopamine receptor subtypes, with D3R and D1R comprising the top 2. The screening also identified some other GPCRs in the 5-HT and α-adrenergic receptor families that bind A69 (Table S2 and Fig. S7), perhaps due to the flexibility properties of this compound as discussed previously.

To test if the non-catechol chemotype of A69 provides an advantage over catechol chemotypes on pharmacokinetics (PK) properties, we analyzed the brain concentrations of A69 following a 30 mg/kg intraperitoneal injection. A69 exhibited sustained high brain concentrations of over 10 μM for the first hour post-injection with an estimated half-life of 1 h (Fig. 6*G*), indicating strong stability and blood–brain barrier penetration.

## Discussion

Here, using virtual computer-screening, we demonstrate that currently available D1R cryo-EM structures are sufficient to identify novel chemotypes for D1R as potential anti-Parkinsonian drugs. Over 1 week’s computational time, we completed a virtual screen of 1 million ZINC database compounds (31) using a standard desktop computer, with hits rapidly acquired from linked commercial vendors at an average cost of $55 per compound. From a physical sub-library of 43 purchased hits, we developed 8 confirmed hits. Performance-wise, the βarr-biased hit was optimized through medicinal chemistry to a lead compound with a Ki value of 86.9 nM, which is stronger than commercial drugs such as dopamine, apomorphine, pergolide and rotigotine (27, 30, 32). Such affinity to D1R is also on par with the lead compounds initially reported by Pfizer after their high-throughput screening and medicinal chemistry optimization (7). This streamlined approach, in which virtual screening becomes the initial step in the discovery process, facilitated our drug development and discovery by minimizing costs and resources. This approach is particularly well-suited for small academic laboratories with time and personnel constraints, orphan or conventional targets, and limited budgets.

In this research, we discovered a novel βarr-biased agonist, A69, at the D1R. In contrast to catechol ligands, which possess low *in vivo* half-lives of minutes (4, 7), A69 demonstrated a much-improved *in vivo* half-life of 1 h. As a potential treatment for PD, A69 with βarr-biased agonism could desensitize the hypersensitized D1R, which has been proposed as the main cause for dyskinesia (33). Arrestin activity has been shown to promote *L*-DOPA-induced recovery for locomotion, while reducing dyskinesia side effects (13). However, it is unclear whether arrestin activity alone is sufficient for driving locomotor responses, an important issue requiring further investigation.

Besides βarr-biased agonism at the D1R, A69 was identified as a D3R antagonist. Interestingly, D3R antagonism has been observed to reduce dyskinesia by modulating the activity of D1R neurons, potentially through direct D1R-D3R interactions (34). Notably, through PDSP screening, A69 was found to bind α2B/2C adrenoceptors, with a Ki for α2B of 459 nM. Antagonists at the α2B/2C adrenoceptor are reported to reduce the severity of LID and to prolong the anti-akinetic effects of single *L*-DOPA doses (35, 36). Together, the unique signaling properties of A69 may reduce LID through multiple distinct mechanisms.

Additionally, we identified A82R as an antagonist at the D1R. Even though some other D1R antagonists, including SCH-23390, are technically non-catechols, their structures are very similar to catechol ligands and, as a result, they possess the same pharmacokinetic limitations (37). By contrast, A82R has a very different chemotype that could provide a much-improved pharmacokinetic profile. As D1R antagonism has been shown to be effective at reducing *L*-DOPA-induced dyskinesia (38), A82R could mitigate this side effect while maintaining its therapeutic effects as mediated through the D2 family receptors. Because D1R antagonism may reduce the therapeutic effects of *L*-DOPA, the dosing and timing of treatment require careful planning to realize its anti-dyskinesia effects without loss of its therapeutic potential.

To our surprise, A82R also demonstrated a strong serotonin transporter (SERT) binding affinity of 9.46 nM, which is notably over 100-fold stronger than its affinity at other neurotransmitter transporters, including the dopamine transporter (DAT) or the norepinephrine transporter (NET).

Due to Pfizer’s optimized series of G protein-biased agonists, we elected not to further pursue our similar G protein-biased hits. However, from a structural perspective, compound A6 only occupied the deep orthosteric pocket, which made it likely to activate all dopamine receptors through similar ligand-receptor interactions. As a result, A6 could be optimized into a dual D1/D2 partial agonist, which potentially could treat PD without the need for *L*-DOPA therapy. This D1/D2 partial agonism could mitigate the risks associated with the overactivation of these receptors and dyskinesia. As a result, A6 could represent another virtual screening hit that may be interesting to optimize and investigate.

Despite striking similarities at the orthosteric site (27), we were able to identify an antagonist with strong D1 family to D2 family selectivity, but we were unable to isolate a completely selective agonist for D1R through virtual screening. This finding was consistent with previous findings of selective antagonists for dopamine receptors by other methods (4), but the discovery of selective agonists remains challenging (27). Interestingly, similar challenges exist for adrenergic receptors (39), histamine receptors (40) and muscarinic receptors (41), likely due to the requirement of conformational adaptability for agonists to induce receptor activation. Alternatively, we recently demonstrated that an allosteric site close to the intracellular loop 2 (ICL2) has great potential for distinguishing dopamine receptor subtypes (26), The antagonists and βarr-biased agonists we discovered in the virtual screening campaign form what we term—a C-shaped binding mode. Their structural similarities suggest that this type of D1R interaction inhibits G protein pathway activation. Interestingly, the non-catechol chemotype that Pfizer discovered forms a distinct linear-shaped binding mode that extends from the dopamine binding region to the Lys81 residue in the extracellular binding pocket. Moreover, extensive SAR work with the Pfizer compounds produced only G protein-biased ligands and balanced agonists (22, 23). Together, these studies suggest that orthosteric biased signaling may be a consequence of a unique binding mode and that the C-shaped mode we identified suggests a template for developing D1R βarr-biased ligands.

Our findings also demonstrate the C-shape binding mode represents a novel strategy to achieve D1 family over D2 family selectivity. Recent studies have shown the orthosteric pockets of different dopamine receptors are remarkably similar (27). We have observed their sequence diversity can only be found in the ECL2 and the EBP regions of the orthosteric pocket of the hD1R. The Pfizer compounds, shown to be D1R family-selective over hD2R (7), likely achieved this feature by engaging the EBP region. In our investigations, the C-shaped binding mode engages the ECL2 region of D1R. The flexible C-shaped lead A69 is adaptable enough to bind alternative dopamine receptors, whereas the rigid lead A82R has strong selectivity for the hD1R family. This latter result is consistent with our hypothesis that D1R selectivity can be achieved through engaging ECL2.

**Figure 7:**
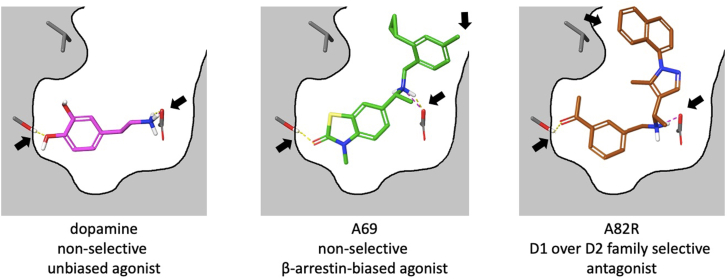
**Information theoretic limits on dopamine receptor agonist selectivity.** Dopamine (*left*) possesses two strong contact points (*black arrow*) and a weak, conserved hydrophobic interaction. The βarr-biased lead A69 (*middle*) introduces additional contact points but is flexible, leading to accommodations at D2 family receptors. The antagonist lead A82R (*right*) can also introduce additional contact points by engaging non conserved residues or extended binding regions, increasing (n) and enabling subtype selectivity even when agonist selectivity is structurally prohibited.

In the case of dopamine, a structural explanation for its long-recognized lack of D1–D5 subtype selectivity emerges naturally when viewed through an information-theoretic lens. Dopamine engages the aminergic GPCR orthosteric pocket through only two independent, discriminative contact points: as represented in the D1R by the protonated amine–Asp^3.32^ salt bridge and the catechol–Serine^5.42^ hydrogen bonding motif. These two interactions define the entire set of microstates available to the ligand (Fig. 7).

Because each independent ligand contact point can be either present or absent, the number of distinct non-empty microstates is given by (2^n^-1), where (n) is the number of independent interactions (14). The corresponding information capacity is therefore H = log_2_(2^n^-1). Although dopamine also participates in a weak aromatic/hydrophobic packing interaction, this contact is highly conserved across aminergic GPCRs and contributes only marginally to (n). As a result, dopamine’s effective (n) remains approximately 2, allowing at most three resolvable microstates, H is approximately equal to 1.6 bits. This capacity is insufficient to uniquely encode the five receptors in the D1–D5 family.

This limitation has direct functional consequences. Because agonists must stabilize the active state ensemble using the same limited set of contacts as the endogenous ligand, dopamine-like agonists cannot introduce additional bits of discriminative information. Subtype selective agonism is therefore structurally unattainable. Antagonists, however, are not bound by the endogenous pharmacophore. They can exploit non-conserved residues, extended orthosteric regions, extracellular loops, or secondary pockets, effectively increasing (n), expanding the microstate space, and raising the bit capacity beyond the limit imposed on dopamine. This distinction explains why subtype-selective dopamine antagonists have been successfully developed, whereas subtype-selective agonists have remained elusive despite decades of medicinal chemistry effort.

From an agonist-bound hD1R cryo-EM structure, we identified antagonists, as well as G protein and βarr-biased ligands. One interpretation of our results is that the orthosteric pocket of the hD1R undergoes minimal conformational changes upon ligand binding, but such conformational changes become selectively amplified by the available transducer. This explanation supports previous findings that balanced or “neutral” agonists and βarr-biased ligands have essentially identical binding modes that induce similar remodeling effects within the orthosteric site (42). Our data provide further support for this model in that the orthosteric pocket is nearly identical both in agonist- and antagonist-bound hD1R structures.

In summary, we have discovered a novel non-catechol D1R antagonist and a βarr-biased agonist through the robustness and utility of virtual screening. These compounds are characterized by a novel C-shaped binding mode that supports interactions with the ECL2 region of the D1R. Our novel non-catechol ligands present a unique opportunity to alleviate dyskinesia associated with PD.

## Experimental procedures

### Cell culture and transfections

Human embryonic kidney HEK-293T cells were obtained from the American Type Culture Collection. U2OS cell lines stably expressing the target receptor and GFP-tagged βarr2 were made by our laboratory (7, 8). Cells were maintained in DMEM (Gibco) supplemented with 10% fetal bovine serum (FBS; Sigma-Aldrich) and 1% antibiotic antimycotic solution (#A5955; Sigma-Aldrich) at 37 °C in a humidified atmosphere with 5% CO_2_. Transfections were conducted using a standard calcium phosphate method (12).

### Molecular docking

Molecular docking was performed using the Maestro platform (Schrodinger). Structures of the hD1R and hD2R with PDB codes 7JVQ (43) and 8IRS (27) were obtained from the Protein Data Bank. Heterotrimeric G proteins were removed, and the receptor-ligand complexes were prepared using the Protein Preparation Wizard. Receptor grids were generated through the Glide Receptor Grid Generation function and centered on the orthosteric site with the grid dimensions approximating the binding pocket of apomorphine or rotigotine from the corresponding resolved structure. Ligands for docking were generated and processed using the LigPrep function with the OPLS_2005 force field. An FDA-approved drug library was obtained from the ZINC database and processed using the LigPrep function, enabling the generation of enantiomers and tautomers. Docking simulations were then performed using Glide's Ligand Docking module, with the receptor grid and pre-processed ligands as inputs. The docking protocol employed flexible ligand sampling in standard precision (SP) mode.

### Virtual screening

A dataset of 6 million 3-D compound structures was sourced from the ZINC database, filtered to include compounds with molecular weights between 250 to 400, logP values less than 5, and designated as "in-stock" for commercial availability. A random sample of approximately 1 million compounds was selected as a starter set. These structures were then screened virtually by docking into the receptor grid, using Glide’s high-throughput virtual screening (HTVS) mode. Compounds with a docking score of −7 or better were retained, narrowing the pool to approximately 20,000 compounds. These compounds were next processed using LigPrep to maintain stereochemistry while generating potential ionization states within a pH range of 6 to 8. The refined set of ligands underwent a second round of virtual screening in SP mode. After applying a docking score threshold of −8, the top 1439 compounds were clustered using the Tanimoto coefficient (0.3 threshold) in RDKit, yielding 266 clusters. The highest-scoring compound from each cluster was manually reviewed for its stability, toxicity, and pharmacokinetic profile. Finally, 43 promising compounds in their racemic mixture form were ordered from MolPort (Latvia) for functional assay testing.

### Competition radioligand binding assay

HEK-293T cells were seeded into 10-cm dishes at 5 × 10^6^ cells/dish and were subsequently co-transfected with 5 μg of hD1R and 5 μg of pcDNA3.1 using calcium-phosphate precipitated transfection. Forty-eight hours post transfection, cells were washed once and harvested in PBS. Cells were centrifuged at 1000*g* for 10 min. Supernatants were removed and the cell pellets were chilled on ice for 5 min before being resuspended and homogenized in 2 ml of hypotonic buffer (50 mM Tris HCl, pH 7.4). The homogenate was centrifuged at 21,000*g* for 20 min in the cold room, and the membrane pellet (precipitate) was either immediately used or stored at −80 °C until later assay.

The membrane pellet was pipetted at 20 μg of protein/well into a round-bottom 96-well plate. To each well was added radioligand ([^3^H]SCH-23390 for D1R and D5R at 1.3 nM, [^3^H]Raclopride for D2S and D3R at 2.5 nM), and the test compound at the indicated concentrations in a total volume of 100 μl. The equilibrium binding reactions proceeded for 1 h at room temperature on a shaker and were terminated by vacuum filtration over 0.3% PEI-soaked GF/B filters using a 96-well Brandel harvester, followed by four washes with wash buffer (50 mM Tris HCl, pH 7.4). The filters were collected, and Bio-Safe II Complete Counting Cocktail (RPI, Mt. Prospect) was added to the vial and the bound radioactivity was counted using a liquid scintillation counter.

### GloSensor cAMP accumulation assay

HEK-293T cells were seeded in 6-well plates at 7.5 × 10^5^ cells/well and were co-transfected with 200 ng of hD1R and 3 μg of GloSensor-22F (Promega) using calcium-phosphate transfection. Twenty-four h post-transfection, cells were plated -into clear-bottom, white-walled, 96-well plates pretreated with Poly-*D*-Lysine at 50,000 cells/well in “BRET media” - clear minimum essential medium (Gibco) supplemented with 2% FBS, 10 mM HEPES, 1x GlutaMax, and 1x antibiotic-antimycotic solution (Sigma-Aldrich). The following day, media were removed, and cells were incubated at room temperature with 25 μl of HHBSS buffer (Hanks’ balanced salt solution (Gibco) supplemented with 20 mM HEPES containing 8 μM D-luciferin for 2 h. Twenty-5 μl of HBSS-HEPES buffer was added to each well, and the cells were treated for 5 min with either vehicle (HHBSS) or the indicated concentration of test drug/compound in a 40 μl volume. Cells were then stimulated with 10 μl of dopamine at the indicated final concentrations. Luminescence was measured using a microplate reader (CLARIOstar; BMG Labtech) 10 min after dopamine or drug/compound stimulation. Dose–response curves were generated with GraphPad Prism 9.

### BRET β-arrestin-2 recruitment assay

HEK-293T cells seeded in 6-well plates at 7.5 × 10^5^ cells/well were co-transfected with 100 ng of hD1R-Rluc8, 1.5 μg of Venus-βarr2 and 1 μg pcDNA3.1 using calcium-phosphate transfection. Twenty-four h post-transfection, cells were plated into clear-bottom, white-walled 96-well plates pretreated with Poly-D-Lysine at 50,000 cells/well in “BRET media” - clear minimum essential medium (Gibco) supplemented with 2% FBS, 10 mM HEPES, 1x GlutaMax, and 1x antibiotic antimycotic solution (Sigma). The following day, media were removed, and cells were incubated at room temperature with 50 μl of HHBSS buffer (Hanks’ balanced salt solution (Gibco) supplemented with 20 mM HEPES) containing 3 μM coelenterazine-H (NanoLight) for 15 min. Cells were treated for 5 min with either vehicle (HHBSS) or the indicated concentration of test drug/compound in a 40 μl volume. Cells were then stimulated with 10 uL of dopamine at the indicated final concentrations. Luminescence at 475-30 nm (donor) and 535-40 nm (acceptor) were assessed using a microplate reader (CLARIOstar; BMG Labtech) 10 min after treatment. BRET ratios were calculated as acceptor signals divided by donor signals. Dose response curves were generated with GraphPad Prism 9.

### Confocal receptor translocation assay

The confocal receptor translocation assay was previously described (12). Briefly, U2OS cells stably expressing βarr2-GFP and various GPCRs were seeded into black, optical-bottom 96-well plates (Nunc) at a density of 20,000 cells/well in Opti-MEM (Gibco) supplemented with 2% FBS. After 24 h, the media were removed and replaced with 60 μl of Opti-MEM. Cells were then treated for 40 min with either vehicle (Opti-MEM) or the indicated compounds added in 40 μl and incubated in a humidified chamber (5% CO_2_, 37 °C). Treatments were terminated with 33 μl of 4% paraformaldehyde and further incubation for 20 min at room temperature. Fixed cells were either stored at 4 °C or immediately imaged for βarr2-GFP translocation using a Zeiss LSM 510 Meta confocal microscope.

### Mouse pharmacokinetic study

The mouse pharmacokinetic study was performed at BioDuro-Sundia and approved by the Shanghai BioDuro-Sundia Institutional Animal Care and Use Committee (IACUC) under protocol IACUC #BD-202501003. A69 was dissolved with 5% DMSO, 95% Tween-80 saline solution (10% Tween-80 in saline) to a final concentration of 6 mg/ml. Male CD-1 mice aged between 6 and 8 weeks were injected with 30 mg/kg of A69 through intraperitoneal injections. Mice were euthanized with carbon dioxide at 30 min, 1 h, 2 h or 4 h post-injection (n = 3 for each time point), and the brains were collected and homogenized. A69 concentration in the brain homogenates was measured by LC/MS/MS.

### Synthesis of A69 and A82R

Synthesis of the hD1R orthosteric ligands A69 and A82R are described in detail in the Supporting Information. ^1^H NMR spectra were recorded using a 400 MHz spectrometer, with compounds dissolved in DMSO. HPLC analyses were performed with two methods. Method 1: Analysis was performed on a Shimadzu Prominence-I LC-2030C 3D Plus instrument. A 5.5-min gradient of 5 to 95% acetonitrile in water (containing 0.05% ammonium hydroxide) was used at a flow rate of 1.5 ml/min. A Waters X-bridge BEH C_18_ column (3 μm, 3 mm × 30 mm) was used at a temperature of 45 °C. Method 2: Analysis was performed on a Shimadzu LCMS-2020 instrument. A 1.8-min gradient of 5 to 95% acetonitrile in water (containing 0.05% ammonium hydroxide) was used with a 3-min run time at a flow rate of 1.0 ml/min. A Waters X-bridge C_18_ column (3.5 μm, 2.1 mm × 50 mm) was used at a temperature of 50 °C. The chiral purity of compounds was analyzed using supercritical fluid chromatography (SFC) on a SHIMADZU LC-30AD system. The method employed was IB N-5-40%D-2.5, using a DAICEL IB N-5 column (4.6 mm × 250 mm, 5 μm particle size). The mobile phase consisted of a mixture of CO_2_ and methanol (MeOH) in a 60:40 ratio, with 0.1% ammonia (7 M in MeOH) as an additive. The flow rate was set to 2.5 ml/min, and the column oven was maintained at 40 °C throughout the analysis. Purity was determined based on HPLC chromatograms using both Method 1 and Method 2, and SFC analysis. All compounds reached at least 95% purity. Mass determination was performed using a Shimadzu LCMS-2020 with electrospray ionization in the positive mode. The ^1^H NMR spectra, HPLC chromatograms, SFC chromatograms, and mass spectra are included in the Supporting Information.

## Data availability

All data generated or analyzed during this study are included in this published article, its supplementary information files and the GitHub repository (https://github.com/Barak-Group/D1R-orthosteric-virtual-screening.git).

## Supporting information

This article contains supporting information.

## Conflict of interest

The authors declare the following financial interests/personal relationships which may be considered as potential competing interests: The Duke Office for Translation and Commercialization has filed a provisional patent application covering composition of matter described in this manuscript. The authors are affiliated with Duke University and may be listed as inventors if this patent is pursued.
