# Supplementary material for: Structure-guided discovery of non-catechol dopamine D1 receptor ligands with biased agonism and antagonism
Source: J Biol Chem. 2026 Apr 27;302(6):113076. doi: 10.1016/j.jbc.2026.113076 (PMC13218150; doi:10.1016/j.jbc.2026.113076)
Supplement: Supplementary material [file mmc1.pdf]

Supporting Information

Structure-guided Discovery of Non-catechol Dopamine D1 Receptor Ligands with Biased Agonism and Antagonism

Yang Zhou,[a] William C. Wetsel,[b] Alem W. Kahsai,[c] Steven H. Olson[d,] and Lawrence S. Barak[a,]**

[a]Department of Cell Biology, Duke University Medical Center, Durham, NC 27710;

[b]Department of Psychiatry and Behavioral Sciences, Duke University Medical Center, Durham, NC 27710;

[c]Department of Medicine, Duke University Medical Center, Durham, NC 27710; and

[d]Conrad Prebys Center for Chemical Genomics at Sanford Burnham Prebys Medical Discovery Institute, La Jolla, CA 92037, USA;

**To whom correspondence may be addressed. Email: lawrence.barak@duke.edu, solson@sbpdiscovery.org.*

Table of contents

Synthesis of A69……..……………………………………………………..S1
Synthetic scheme………………………………………………………...S1
Synthetic procedures…………………………………………………….S1
^{1}H NMR spectra…………………………………………………….S4
HPLC chromatograms…………………………………………………...S5
Mass spectrum…………………………………………………………...S5
SFC analysis……………………………………………………………..S6
Synthesis of A82R…………………………..……………………….....S7
Synthetic scheme………………………………………………………...S7
Synthetic procedures…………………………………………………….S7
^{1}H NMR spectra………………………………………….………..S11
HPLC chromatograms…………………………………….……………S11
Mass spectrum…………………………………………………………S12
SFC analysis……………………………………………………………S12
Supplementary Figure 1………………………………………………S13
Supplementary Figure 2………………………………………………S14
Supplementary Figure 3………………………………………………S15
Supplementary Figure 4………………………………………………S16
Supplementary Figure 5………………………………………………S17
Supplementary Figure 6………………………………………………S19
Supplementary Figure 7………………………………………………S20
Supplementary Table 1………………………………………………..S24
Supplementary Table 2………………………………………………..S24

Synthesis of A69

Synthetic Scheme of A69

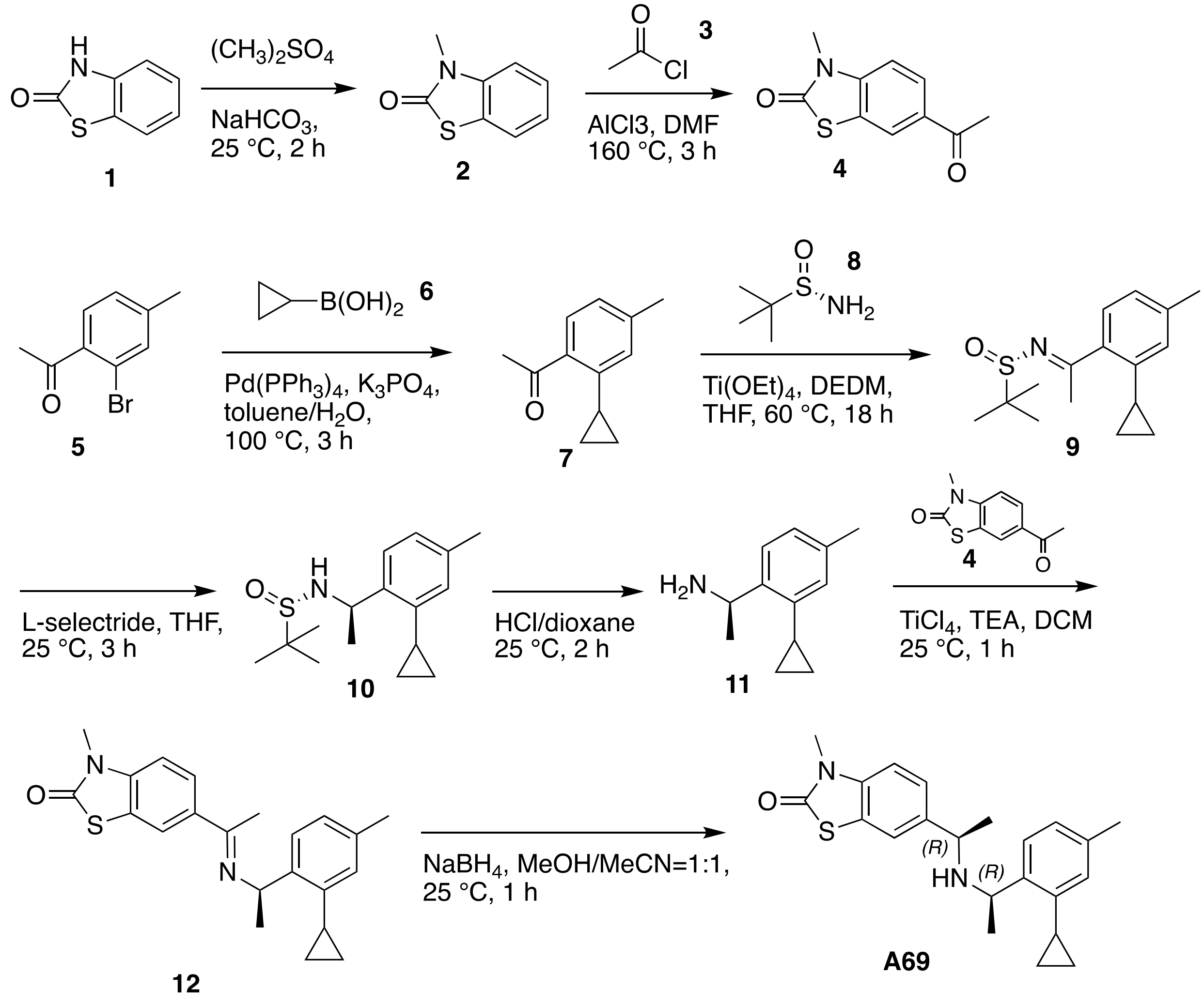

Synthetic procedure:

Step 1： 3-methylbenzo[d]thiazol-2(3H)-one (2)

To a solution of 3H-1,3-benzothiazol-2-one (**1**, 2 g, 13.2 mmol) and K_2CO_3 (5.47 g, 39.6 mmol) in DMF (20 mL) was added MeI (2.81 g, 19.8 mmol). The reaction mixture was stirred at 60 ºC for 3 h. The mixture was diluted with water (100 mL) and extracted with EtOAc (50 mL x 3). The combined organic layers were dried over Na_2SO_4, filtered and concentrated. The residue was purified by flash chromatography column (eluent EtOAc in PE = 0-20%) to give the **Cpd 2** (2 g, 83% yield) as pale yellow solid.

^{1}H NMR (400 MHz, DMSO-d_6): δ 7.644 (d, J=7.6 Hz, 1H), 7.409-7.371 (m, 1H), 7.306-7.286 (m, 1H), 7.230-7.192 (m, 1H), 3.405 (s, 3H).

Step 2: 6-acetyl-3-methylbenzo[d]thiazol-2(3H)-one (4)

To a solution of 3-methyl-1,3-benzothiazol-2-one (**2**, 1 g, 6.1 mmol) and $FeCl_3$ (5.94 g, 36.6 mmol) in DMF (1 mL) was added acetyl chloride (**3**, 0.72 g, 9.15 mmol). The reaction mixture was stirred at 160 °C for 3 h. The reaction mixture was diluted with water (10 mL) and extracted with EtOAc (10 mL x 3). The combined organic layers were dried over Na_2SO_4, filtered and concentrated. The residue was purified by flash chromatography column (eluent EtOAc in PE = 0-15%) to give the **Cpd 4** (0.8 g, 61% yield) as pale yellow solid.
^{1}H NMR (400MHz, DMSO-d_6): δ 8.310 (d, J = 1.2 Hz, 1H), 7.973 (s, 1H), 7.416 (d, J = 8.4 Hz, 1H), 3.447 (s, 3H), 2.580 (s, 3H).

Step 3：1-(2-cyclopropyl-4-methylphenyl)ethan-1-one (7)

A solution of 1-(2-bromo-4-methylphenyl)ethanone (**5**, 500 mg, 2.34 mmol), cyclopropyl boronic acid (**6**, 262 mg, 3.05 mmol), K_3PO_4 (1743 mg, 8.21 mmol), $Pd(PPh_3)_4$ (271 mg, 0.23 mmol) in toluene/ H_2O (10 mL/1 mL) was heated to 100 °C for 3 h. The mixture was diluted with water (10 mL) and extracted with EtOAc (10 mL x 3). The combined organic layers were dried over Na_2SO_4, filtered and concentrated. The residue was purified by flash chromatography column (eluent EtOAc in PE = 0-10%) to give the **Cpd 7** (400 mg, 88% yield) as yellow solid.
MS (ESI): mass calcd. for $C_{12}H_{14}O$ 174.10, m/z found 175.1 $[M+H]^+$.
^{1}H NMR (400 MHz, $CDCl_3$): δ 7.48 (d, J = 8.0 Hz, 1H), 7.02(d, J = 8.0 Hz, 1H), 6.84 (s, 1H), 2.60 (s, 3H), 2.48 – 2.44 (m, 1H), 2.33 (s, 3H), 1.00 – 0.95 (m, 2H), 0.68 – 0.64 (m, 2H).

Step 4: (S,E)-N-(1-(2-cyclopropyl-4-methylphenyl)ethylidene)-2-methylpropane-2-sulfin amide (9)

A solution of 1-(2-cyclopropyl-4-methylphenyl)ethan-1-one (7, 695 mg, 3.98 mmol), (S)-2-methylpropane-2-sulfinamide (8, 580 mg, 4.78 mmol), titanium ethoxide (1819 mg, 7.97 mmol) and DEDM (268 mg, 1.99 mmol) in THF (12 mL) was stirred at 60 °C for 18 h. The mixture was diluted with water (10 mL) and extracted with EtOAc (10 mL x 3). The combined organic layers were dried over Na_2SO4, filtered and concentrated. The residue was purified by flash

chromatography column (eluent EtOAc in PE = 0-10%) to give **Cpd 9** (483 mg, 39% yield) as yellow oil.

MS (ESI): mass calcd. for $C_{16}H_{23}NOS$ 277.15, m/z found 278.1 $[M+H]^+$.

^1H NMR (400MHz, $CDCl_3$): δ 7.17 (d, J = 7.6 Hz, 1H), 7.01 (d, J = 7.2 Hz, 1H), 6.82 (s, 1H), 2.75 (s, 3H), 2.53 (s, 1H), 2.32 (s, 3H), 1.29 (s, 9H), 0.94 (d, J = 8.4 Hz, 2H), 0.69 (s, 2H).

Step 5: (S)-N-((R)-1-(2-cyclopropyl-4-methylphenyl)ethyl)-2-methylpropane-2-sulfinamide (10)

To a solution of (S)-N-[(1E)-1-(2-cyclopropyl-4-methylphenyl)ethylidene]-2-methylpropane-2-sulfinamide (**9**, 856 mg, 3.08 mmol) in THF (10 mL) was added 1M L-selectride in THF (7.7 mL) dropwise in ice-water bath. The reaction mixture was stirred at 25 °C for 3 h. The mixture was diluted with water (10 mL) and extracted with EtOAc (10 mL x 3). The combined organic layers were dried over Na_2SO_4, filtered and concentrated. The residue was purified by flash chromatography column (eluent EtOAc in PE = 30-50%) to give the **Cpd 10** (765 mg, 79% yield) as colorless oil.

MS (ESI): mass calcd. for $C_{16}H_{25}NOS$ 279.17, m/z found 280.3 $[M+H]^+$.

^1H NMR (400MHz, $CDCl_3$): δ 7.25 (s, 1H), 7.02 (d, J = 7.6 Hz, 1H), 6.87 (s, 1H), 5.25 – 5.23 (m, 1H), 3.31 (s, 1H), 2.30 (s, 3H), 1.53 (d, J = 6.8 Hz, 3H), 1.29 – 1.24 (m, 1H), 1.20 (s, 9H), 0.98 – 0.92 (m, 2H), 0.78 – 0.73 (m, 1H), 0.65 – 0.59 (m, 1H).

Step 6: (R)-1-(2-cyclopropyl-4-methylphenyl)ethan-1-amine (11)

To a solution of (S)-N-[(1R)-1-(2-cyclopropyl-4-methylphenyl)ethyl]-2-methylpropane-2-sulfin amide (**10**, 300 mg, 1.07 mmol) in 1,4-dioxane (2 mL) was added 4M HCl in dioxane (2 mL). The mixture was stirred at 25 °C for 2 h. The mixture was evaporated and washed by PE (10 mL) to give the **Cpd 11** (88 mg, 42% yield) as white solid.

^1H NMR (400MHz, $CDCl_3$): δ 8.60 (s, 2H), 7.54 (s, 1H), 7.01 (s, 1H), 6.91 (s, 1H), 5.09 (s, 1H), 2.28 (s, 3H), 1.64 (s, 3H), 1.25 (s, 1H), 0.92 (d, J = 7.2 Hz, 2H), 0.77 - 0.76 (m, 1H), 0.56 - 0.54 (m, 1H).

Step 7：6-((R)-1-(((R)-1-(2-cyclopropyl-4-methylphenyl)ethyl)amino)ethyl)-3-methylbenzo[d] thiazol-2(3H)-one (A69)

To a solution of 6-acetyl-3-methylbenzo[d]thiazol-2(3H)-one (**4**, 300 mg, 1.44 mmol), (R)-1-(2-cyclopropyl-4-methylphenyl)ethan-1-amine (**11**, 304 mg, 1.73 mmol) in DCM (15 mL) was added TEA (585 mg, 5.79 mmol) and $TiCl_4$ (1M in THF, 2.9 mL, 2.9 mmol) in ice-water bath. The mixture was stirred at 25 °C for 1 h. LCMS showed SM was consumed and **Cpd 12** was detected. The mixture was added ACN (4 mL) and $NaBH_4$ (1095 mg, 28.95 mmol) at 0 °C under nitrogen, then MeOH (4 mL) was added. The mixture was stirred at 25 °C for 1 h. The mixture was quenched with water (30 mL) and was filtered. The filtrate was collected and extracted with EtOAc (30 mL x 3). The combined organic layers were dried over Na_2SO_4, filtered and concentrated. The residue was purified by flash chromatography column (eluent EtOAc in PE = 0-30%) to give the crude product, the residue was purified by reverse column ([H_2O (0.05% FA-H_2O)-ACN]; B%: 5% - 40%, 30 min) to give the **Cpd A69** (122.26 mg, 22% yield) as white solid.

MS (ESI): mass calcd. for $C_{22}H_{26}N_2OS$ 366.18, m/z found 367.3 $[M+H]^+$.

^{1}H NMR (400MHz, DMSO-d_6): δ 7.47 – 7.44 (m, 2H), 7.25 – 7.21 (m, 2H), 7.01 (d, J = 7.2 Hz, 1H), 6.71 (s, 1H), 4.03 – 3.98 (m, 1H), 3.39 (s, 4H), 2.43 (s, 1H), 2.23 (s, 3H), 1.52 – 1.45 (m, 1H), 1.20 – 1.14 (m, 6H), 0.65 – 0.58 (m, 1H), 0.52 – 0.38 (m, 2H), 0.24 – 0.18 (m, 1H).

NMR spectrum:

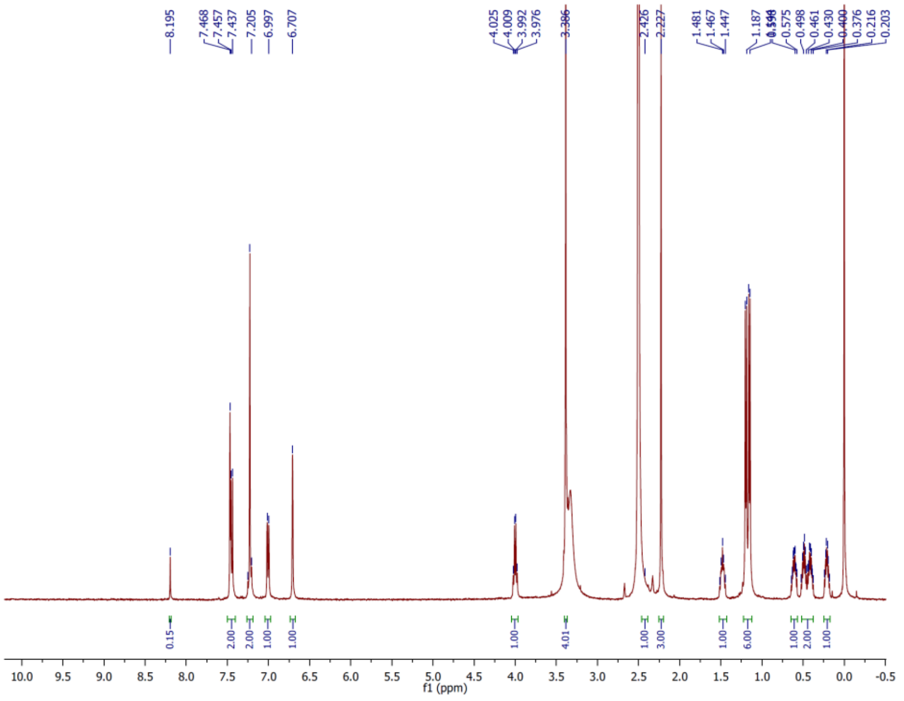

HPLC chromatogram system 1:

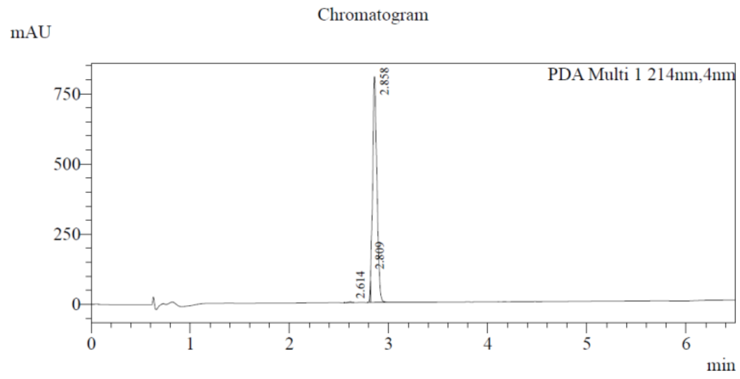

HPLC chromatogram system 2:

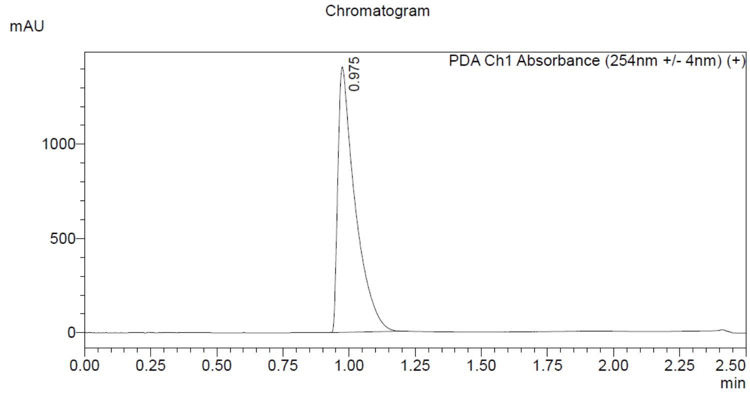

Mass spectrum:

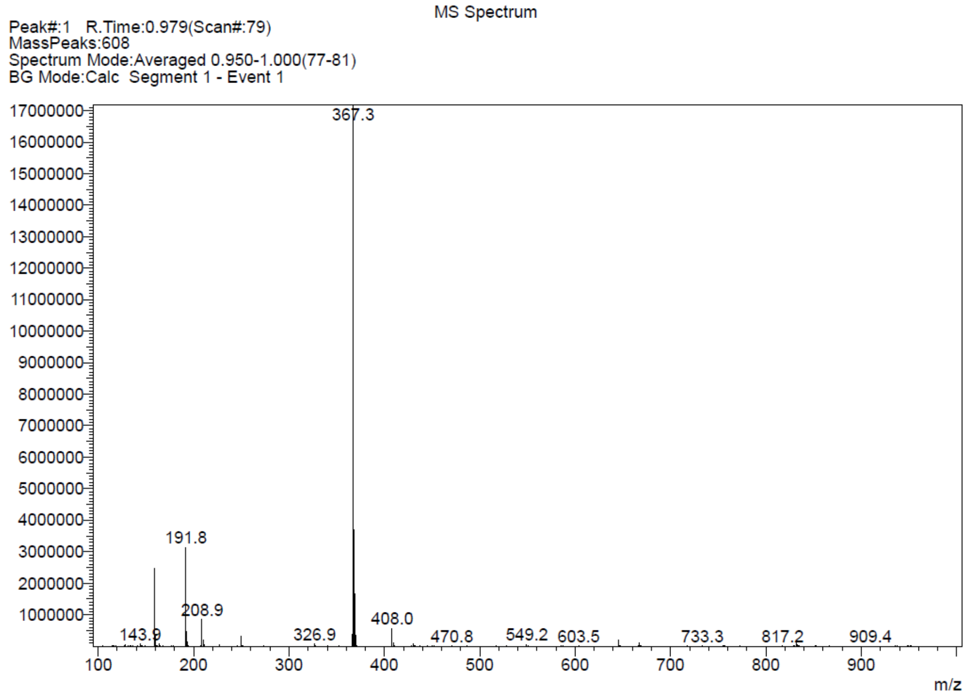

SFC analysis:

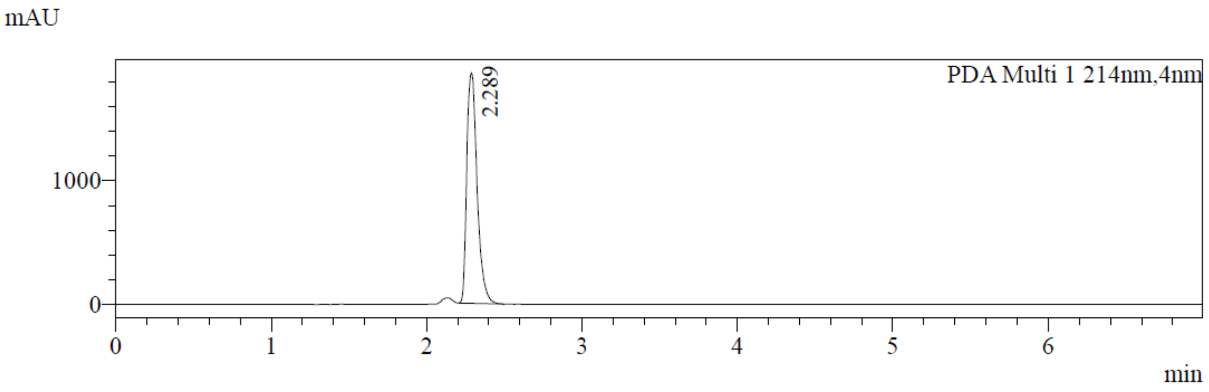

Synthesis of A82R

Synthetic Scheme of A82R

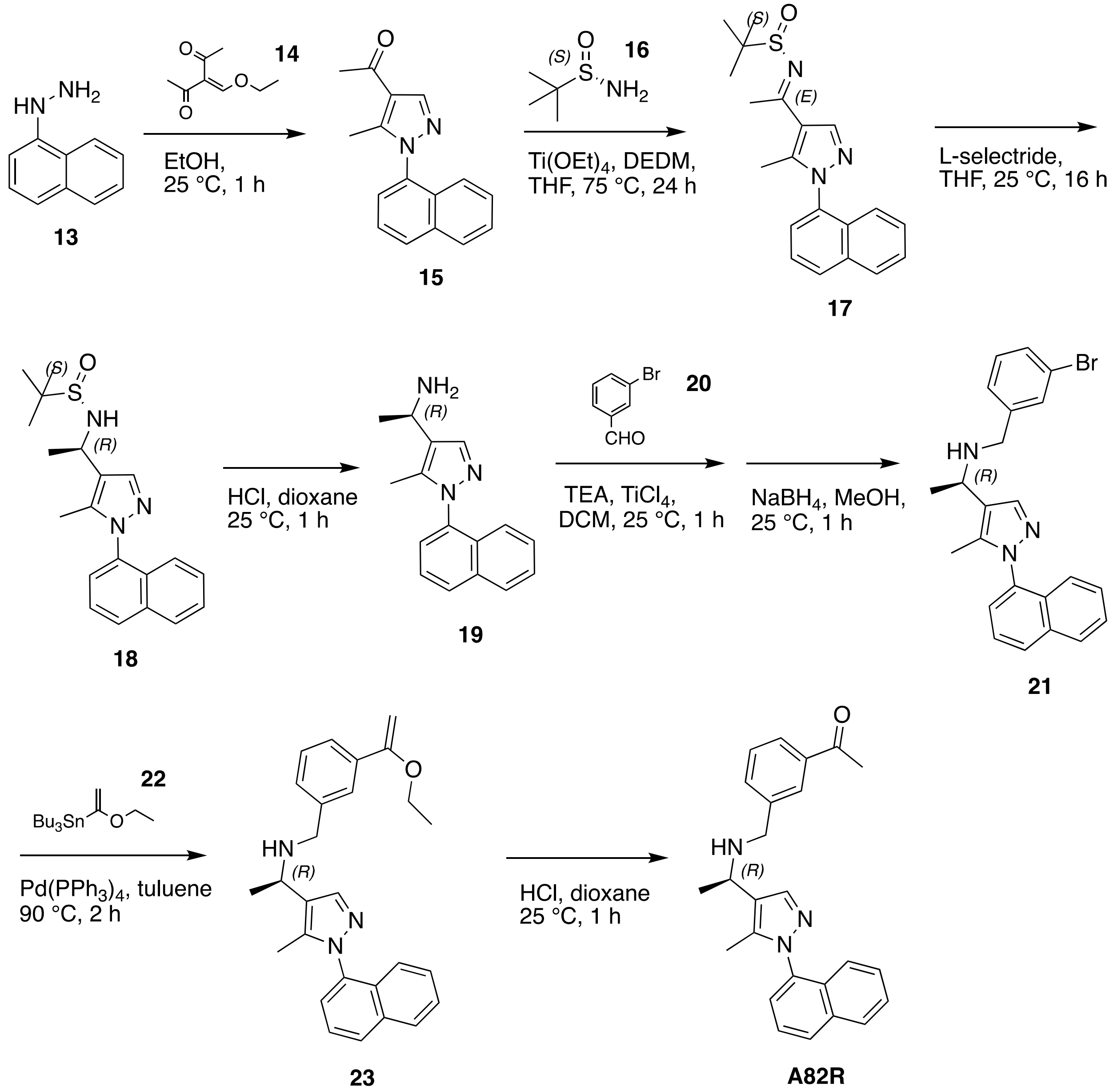

<u>**Synthetic procedure:**</u>

<u>**Step 1： 1-(5-methyl-1-(naphthalen-1-yl)-1H-pyrazol-4-yl)ethan-1-one (15)**</u>

A solution of naphthalen-1-ylhydrazine (**13**, 2 g, 12.6 mmol) and 3-(ethoxymethylene)pentane-2,4-dione (**14**, 2.16 g, 13.86 mmol) in EtOH (30 mL) was stirred at 25 ºC for 1h. The mixture was concentrated under reduced pressure. The residue was purified by flash chromatography

column (eluent EtOAc in petroleum ether = 0-90%) to obtain **Cmp 15** (2 g, 63% yield) as yellow oil.

MS (ESI): mass calcd. for $C_{16}H_{14}N_2O$ 250.11, m/z found 250.9 $[M+H]^+$.

^{1}H NMR (400 MHz, DMSO-d_6): δ 8.36 (s, 1H), 8.19 – 8.11 (m, 2H), 7.71 – 7.56 (m, 4H), 7.14 (d, J = 8.0 Hz, 1H), 3.34 (s, 3H), 2.27 (s, 3H).

Step 2：(S,E)-2-methyl-N-(1-(5-methyl-1-(naphthalen-1-yl)-1H-pyrazol-4-yl)ethylidene) propane-2-sulfinamide (17)

To a solution of (S)-2-methylpropane-2-sulfinamide (**16**, 0.58 g, 4.8 mmol), $Ti(OEt)_4$ (1.82 g, 8 mmol) and diglyme (0.27 g, 2 mmol) in THF (10mL) was added (1-[5-methyl-1-(naphthalen-1-yl)pyrazol-4-yl]ethenone (**15**, 1 g, 4 mmol). The reaction mixture was stirred at 75 ºC for 24 h. The mixture was diluted with water (20 mL) and extracted with EtOAc (10 mL x 3). The combined organic layers were dried over Na_2SO_4, filtered and concentrated. The residue was purified by flash chromatography column (eluent EtOAc in petroleum ether = 25-90%) to give **Cmp 17** (0.63g, 40% yield) as pale-yellow oil.

MS (ESI): mass calcd. for $C_{20}H_{23}N_3OS$ 353.16, m/z found 354.0 $[M+H]^+$.

^{1}H NMR (400MHz, DMSO-d_6): δ 8.33 (s, 1H), 8.18 (d, J = 7.6 Hz, 1H), 8.11 (d, J = 8.0 Hz, 1H), 7.71 – 7.62 (m, 3H), 7.60 – 7.56 (m, 1H), 7.16 (d, J = 8.4 Hz, 1H), 2.73 (s, 3H), 2.32 (s, 3H), 1.20 (s, 9H).

Step 3: (R)-2-methyl-N-(1-(5-methyl-1-(naphthalen-1-yl)-1H-pyrazol-4-yl)ethyl)propane-2-sulfinamide (18)

To a solution (S,E)-2-methyl-N-(1-(5-methyl-1-(naphthalen-1-yl)-1H-pyrazol-4-yl)ethylidene) propane-2-sulfinamide (**17**, 630 mg, 1.78 mmol) in THF (10 mL) was added L-selectride (1M in THF, 5.4 mL, 5.4 mmol) in ice-water bath.The reaction mixture was stirred at 25 ºC for 16 h. The mixture was diluted with water (10 mL) and extracted with EtOAc (10 mL x 3). The combined organic layers were dried over Na_2SO_4, filtered and concentrated. The residue was

purified by flash chromatography column (eluent EtOAc in petroleum ether = 80-90%) to give **Cmp 18** (515 mg, 81% yield) as yellow oil.

MS (ESI): mass calcd. for $C_{20}H_{25}N_3OS$ 355.17, m/z found 356.0 [M+H] $^+$.

^{1}H NMR (400MHz, DMSO-d_6): δ 8.12 – 8.07 (m, 2H), 7.68 – 7.51 (m, 5H), 7.11 (d, J = 8.4 Hz, 1H), 5.31 (d, J = 5.2 Hz, 1H), 4.47 – 4.44 (m, 1H), 1.99 (s, 3H), 1.58 – 1.53 (m, 3H), 1.13 (s, 9H).

Step 4: (R)-1-(5-methyl-1-(naphthalen-1-yl)-1H-pyrazol-4-yl)ethan-1-amine (19)

A solution of (R)-2-methyl-N-(1-(5-methyl-1-(naphthalen-1-yl)-1H-pyrazol-4-yl)ethyl)propane-2-sulfinamide (**18**, 515 mg, 1.44 mmol) in 4M HCl in dioxane (5 mL) was stirred at 25 °C for 1 h. The mixture was evaporated and washed by petroleum ether (5 mL) to give **Cmp 19** (250 mg, 62% yield) as white solid, which was used for next step without further purification.

MS (ESI): mass calcd. for $C_{16}H_{17}N_3$ 251.14, m/z found 253.1 [M+H] $^+$.

^{1}H NMR (400MHz, DMSO-d_6): δ 8.26 (s, 2H), 8.16 – 8.09 (m, 2H), 7.91 (s, 1H), 7.70 – 7.53 (m, 4H), 7.15 (d, J = 8.4 Hz, 1H), 4.50 – 4.43 (m, 1H), 1.61 (d, J = 6.8 Hz, 3H).

Step 5: (R)-N-(3-bromobenzyl)-1-(5-methyl-1-(naphthalen-1-yl)-1H-pyrazol-4-yl)ethan-1-amine (21)

To a solution of (R)-1-(5-methyl-1-(naphthalen-1-yl)-1H-pyrazol-4-yl)ethan-1-amine (**19**, 250 mg, 0.99 mmol) and 3-bromobenzaldehyde (**20**, 368 mg, 1.98 mmol) in DCM (15 mL) was added TEA (805 mg, 7.95 mmol) and $TiCl_4$ (1M in THF, 2 mL, 2 mmol) in ice-water bath. The mixture was stirred at 25 °C for 1 h. The mixture was added $NaBH_4$ (188 mg, 4.97 mmol) at 0 °C under nitrogen. Then the MeOH (5 mL) was added. The mixture was stirred at 25 °C for 1 h. The mixture was quenched with water (15 mL) and was filtered. The filtrate was collected and extracted with EtOAc (10 mL x 3). The combined organic layers were dried over Na_2SO_4, filtered and concentrated. The residue was purified by flash chromatography column (eluent EtOAc in petroleum ether = 50-90%) to give the title compound (250 mg, 51% yield) as yellow oil.

MS (ESI): mass calcd. for $C_{23}H_{22}BrN_3$ 419.10, m/z found 420.3 [M+H] $^+$.

Step 6: (R)-N-(3-(1-ethoxyvinyl)benzyl)-1-(5-methyl-1-(naphthalen-1-yl)-1H-pyrazol-4-yl)eth an-1-amine (23)

A solution of (R)-N-(3-bromobenzyl)-1-(5-methyl-1-(naphthalen-1-yl)-1H-pyrazol-4-yl)ethan-1-amine (**21**, 250 mg, 0.59 mmol), tributyl(1-ethoxyvinyl)stannane (429 mg, 1.18 mmol), $Pd(PPh_3)_4$ (68 mg, 0.059 mmol) in toluene (12 mL) was heated to 90 °C and stirred for 2 h. The mixture was diluted with water (10 mL) and extracted with EtOAc (10 mL x 3). The combined organic layers were dried over Na_2SO_4, filtered and concentrated. The residue was purified by flash chromatography column (eluent EtOAc in petroleum ether = 60-90%) to give the title compound 200 mg, 74% yield) as pale-yellow oil.

MS (ESI): mass calcd. for $C_{27}H_{29}N_3O$ 411.23, m/z found 412.3 $[M+H]^+$.

Step 7: (R)-1-(3-(((1-(5-methyl-1-(naphthalen-1-yl)-1H-pyrazol-4-yl)ethyl)amino) methyl) ph enyl)ethan-1-one (A82R)

A solution of (R)-N-(3-(1-ethoxyvinyl)benzyl)-1-(5-methyl-1-(naphthalen-1-yl)-1H-pyrazol-4-yl)ethan-1-amine (**23**, 200 mg, 0.48 mmol) in 4 M HCl in dioxane (5 mL) was stirred at 25 °C for 1 h. The mixture was evaporated and washed by petroleum ether to give the crude product, and the residue was purified by prep-HPLC (column: Phenomenex luna C18 150 mm x 19 mm x 5 um; mobile phase: [H_2O (0.05% NH_3-H_2O)-ACN]; B%: 50% - 90%, 35 min) to give the title compound **A82R** (14.10 mg, 8% yield) as colorless oil.

MS (ESI): mass calcd. for $C_{25}H_{25}N_3O$ 383.20, m/z found 384.0 $[M+H]^+$.

^{1}H NMR (400MHz, MeOD): δ 8.07 (d, J = 8.4 Hz, 1H), 8.02 – 7.97 (m, 2H), 7.90 (d, J = 7.6 Hz, 1H), 7.81 (s, 1H), 7.65 – 7.46 (m, 6H), 7.14 (d, J = 8.0 Hz, 1H), 3.90 – 3.81 (m, 3H), 2.59 (s, 3H), 1.91 (s, 3H), 1.50 (d, J = 6.4 Hz, 3H).

NMR spectrum

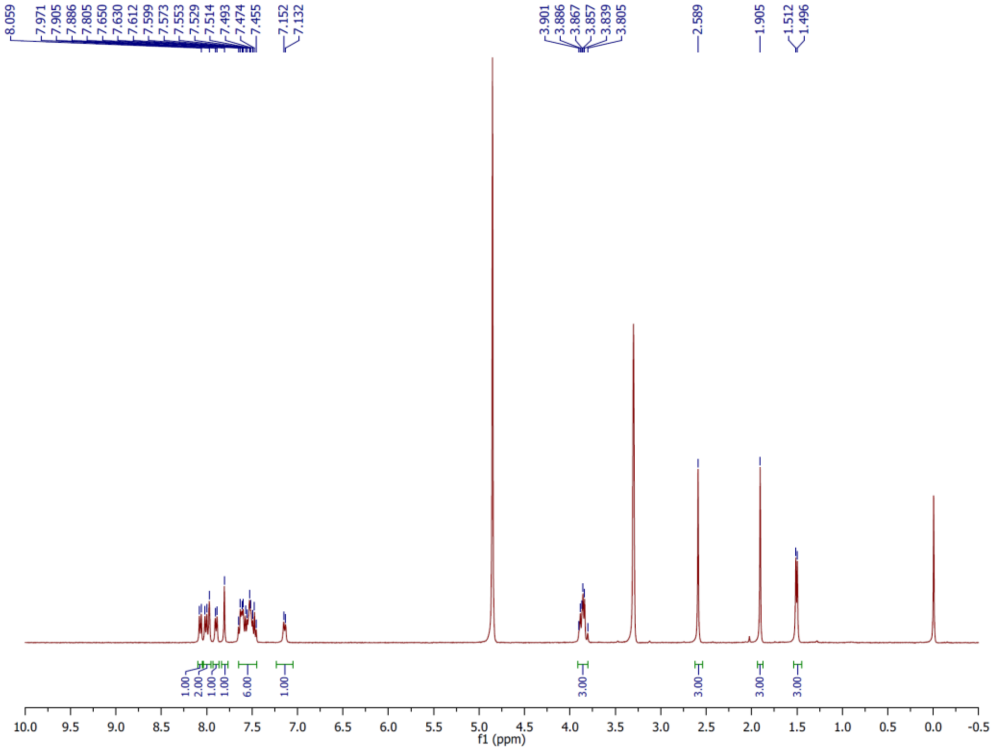

HPLC chromatogram system 1:

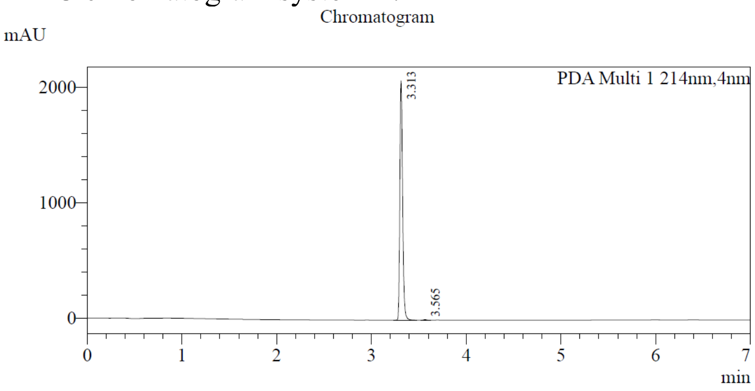

HPLC chromatogram system 2:

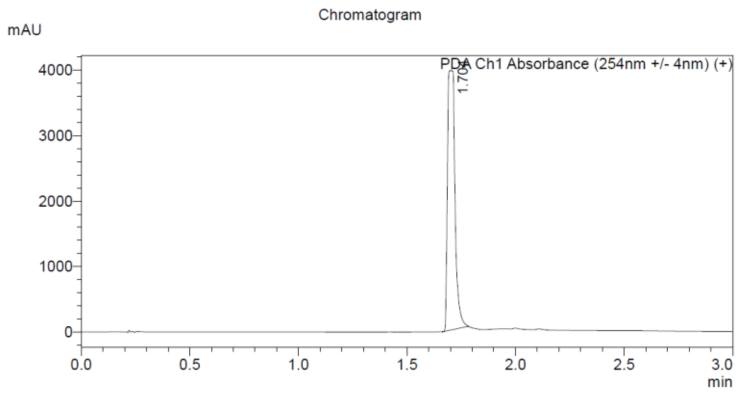

Mass spectrum:

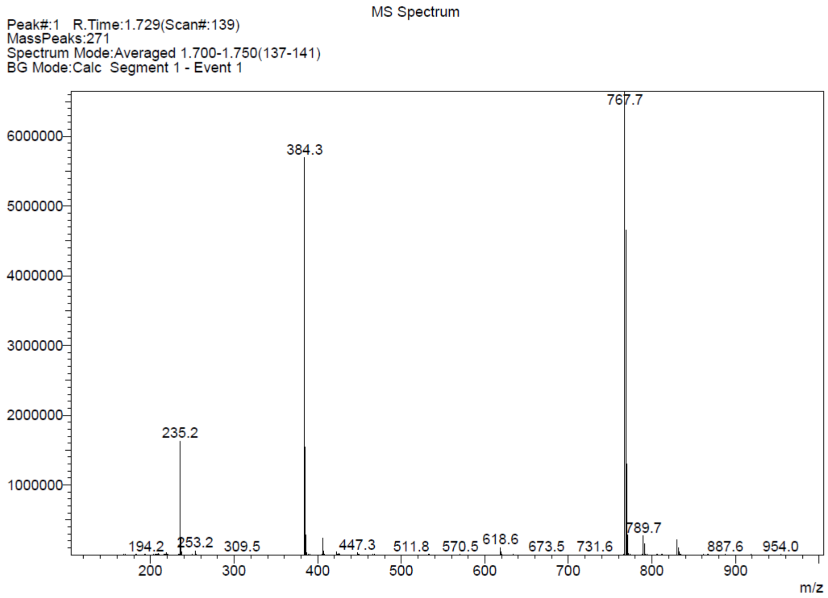

SFC analysis:

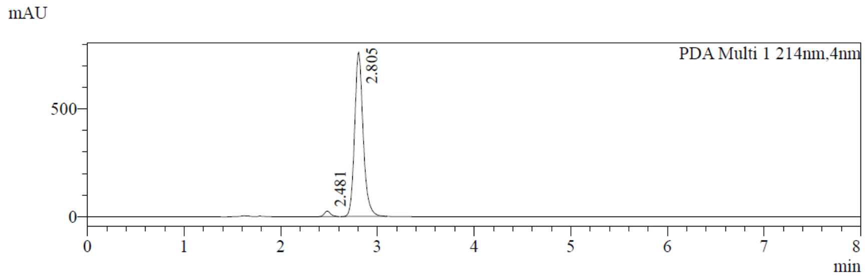

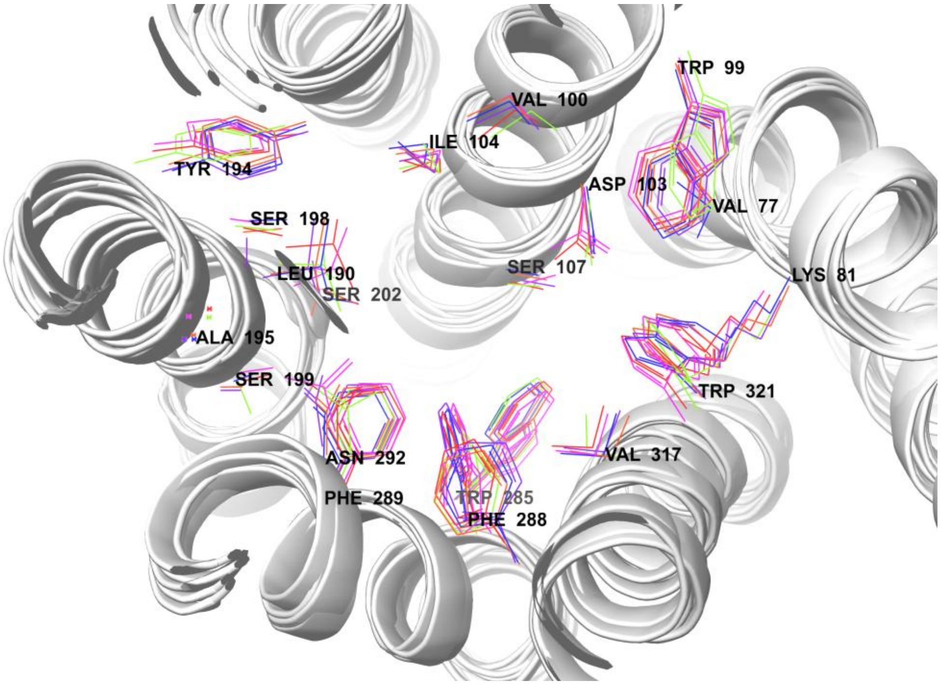

Supplementary Figure 1. Superimposition of six cryo-EM structures of the human dopamine D1 receptor, with key residues pointing towards the orthosteric pocket labelled and colored (7JV5: blue, 7JVP: purple, 7JVQ: orange, 7CKW: red, 7CKX: green, 7CKY: pink).

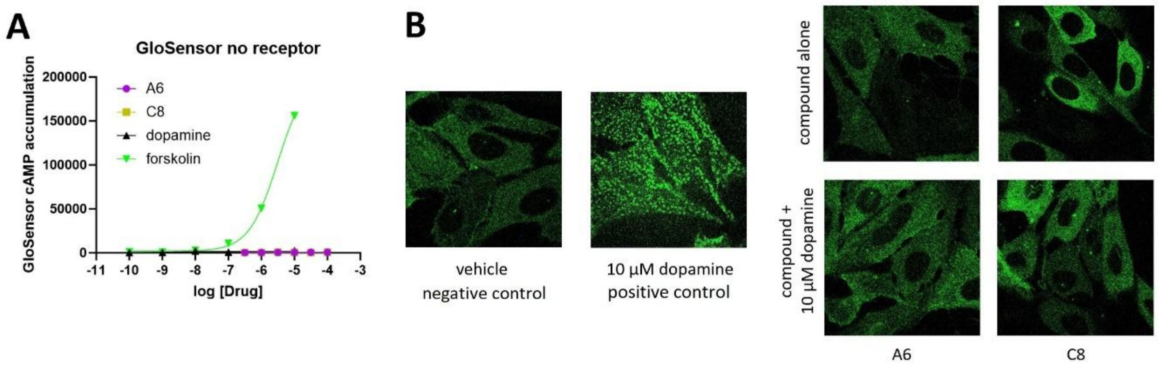

Supplementary Figure 2. Response to Compounds A6 and C8. (A) GloSensor cAMP accumulation is shown in the presence of compounds (A6, C8, dopamine, or forskolin) and in the absence of transfected hD1R. N = 3 independent experiments. Error bars represent ± SD. **(B) βArr-mediated receptor translocation assay** is shown for U2OS cells overexpressing hD1R and βarr2-GFP. For this supplementary figure and the next two (SF3 and SF4), the left fluorescence images labeled vehicle and 10 µM dopamine serve as the negative and positive controls delineating the range of fluorescent aggregate formation. Shown at the right-hand side are panels treated with vehicle or with 100 µM of a respective compound (A6 or C8) alone (upper panel) or in the presence of 10 µM dopamine (lower panel). N = 3 independent experiments.

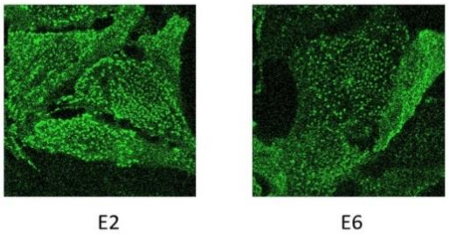

Supplementary Figure 3. βArr-mediated receptor translocation assay is shown for U2OS cells overexpressing hD1R and βarr2-GFP. Shown are panels expressing fluorescence aggregates where cells were treated with 100 μM of a respective compound (E2 - left panel or E6 – right panel) alone. N = 3 independent experiments.

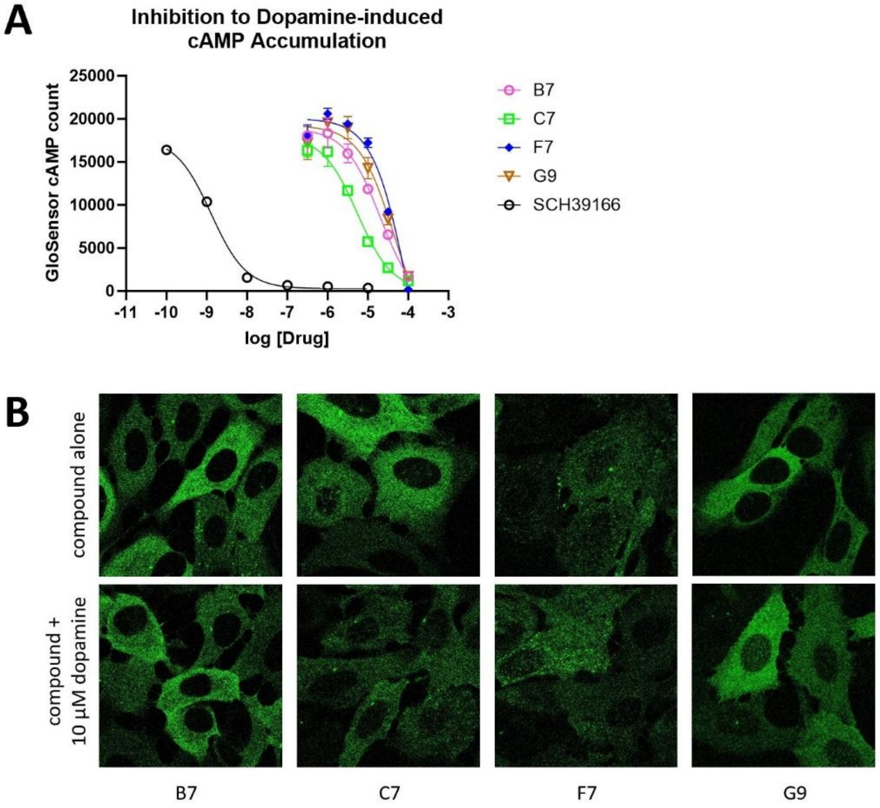

Supplementary Figure 4. Response to Compounds B7, C7, F7, and G9. (A) D1R GloSensor assay measuring the dose-dependence of novel antagonists B7, C7, F7, G9 and control compound SCH39166 at blocking 1 nM dopamine induced cAMP accumulation. N = 3 independent experiments. Error bars represent ± SD, **(B) βArr-mediated receptor translocation assay** is shown for U2OS cells overexpressing hD1R and βarr2-GFP. Shown are panels treated with100 µM of a respective compound (**B7, C7, F7, and G9 left to right**) alone (upper panel) or in the presence of 10 µM dopamine (lower panel). N = 3 independent experiments.

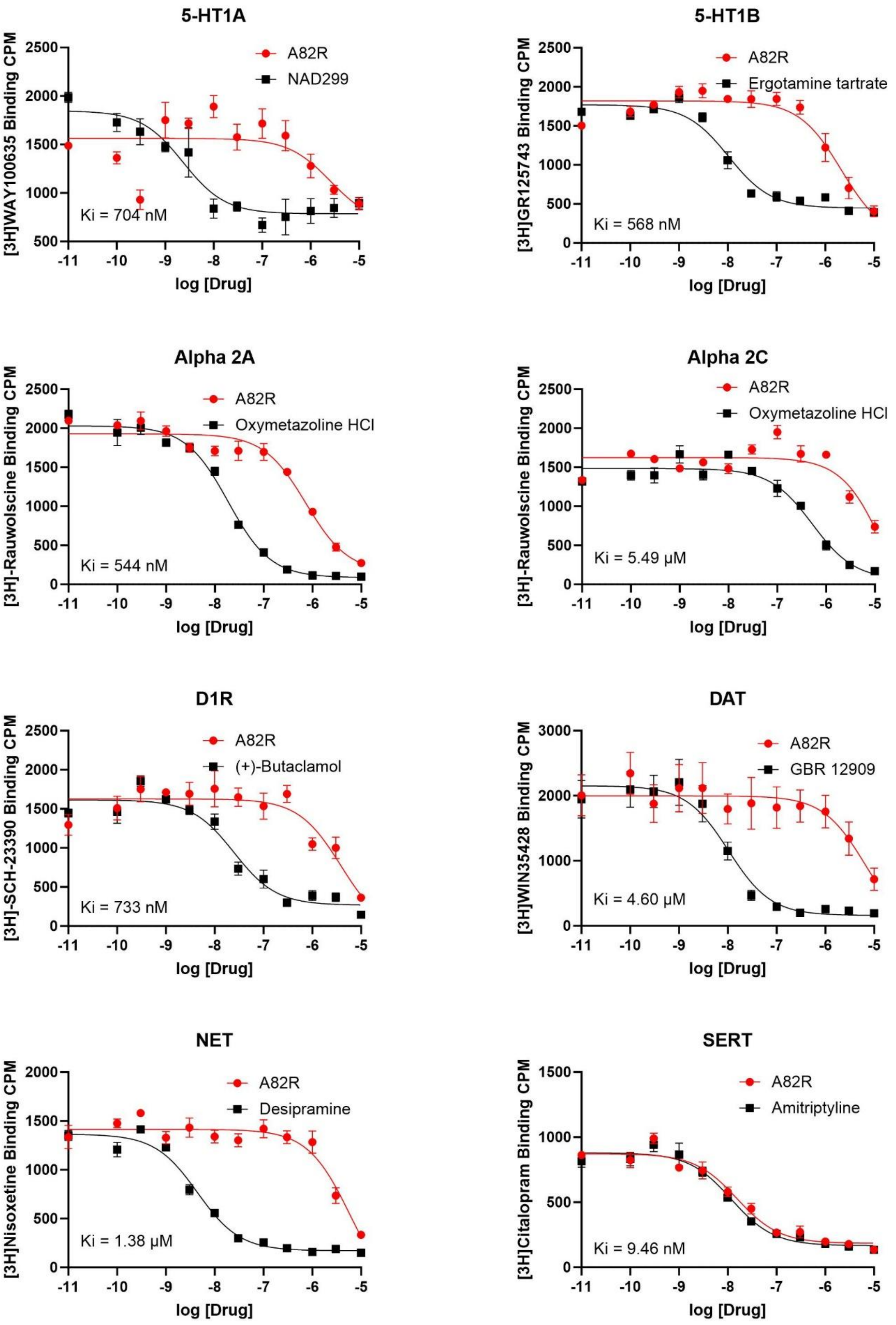
5-HT1A
[3H]WAY100635 Binding CPM
A82R
NAD299
Ki = 704 nM
log [Drug]
5-HT1B
[3H]GR125743 Binding CPM
A82R
Ergotamine tartrate
Ki = 568 nM
log [Drug]
Alpha 2A
[3H]-Rauwolscine Binding CPM
A82R
Oxymetazoline HCl
Ki = 544 nM
log [Drug]
Alpha 2C
[3H]-Rauwolscine Binding CPM
A82R
Oxymetazoline HCl
Ki = 5.49 μM
log [Drug]
D1R
[3H]-SCH-23390 Binding CPM
A82R
(+)-Butaclamol
Ki = 733 nM
log [Drug]
DAT
[3H]WIN35428 Binding CPM
A82R
GBR 12909
Ki = 4.60 μM
log [Drug]
NET
[3H]Nisoxetine Binding CPM
A82R
Desipramine
Ki = 1.38 μM
log [Drug]
SERT
[3H]Citalopram Binding CPM
A82R
Amitriptyline
Ki = 9.46 nM
log [Drug]

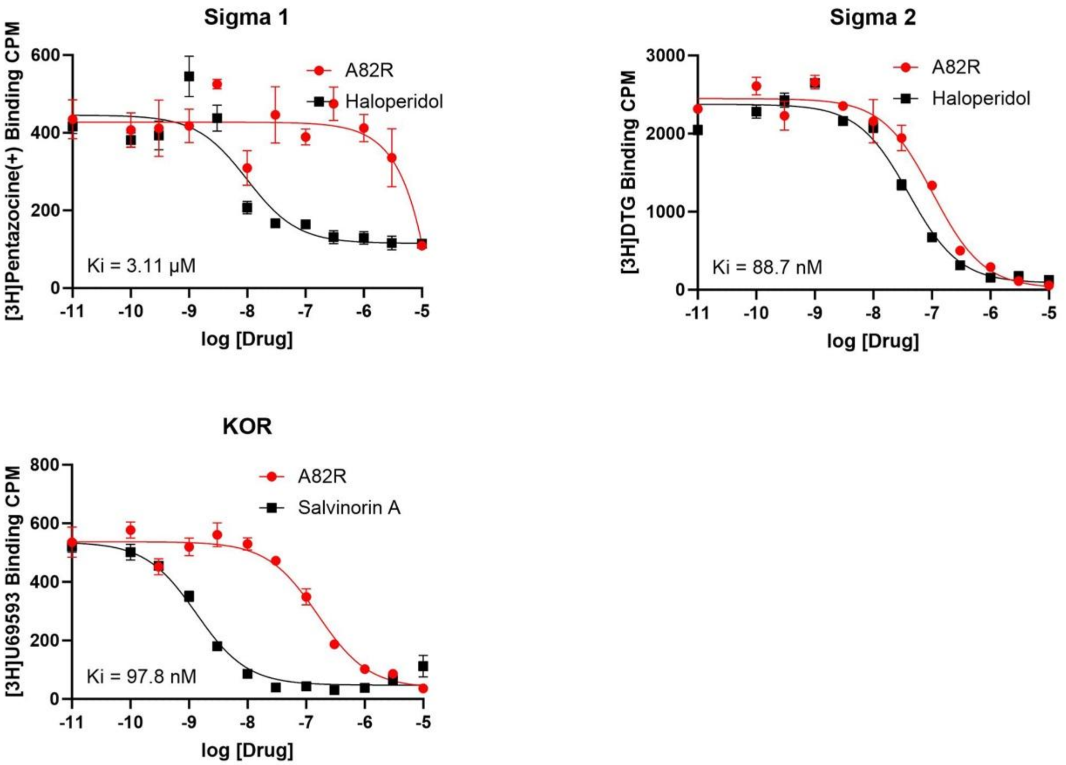

Supplementary Figure 5. Competitive radioligand binding assays testing dose-responses of A82R and control compounds at various receptor targets in the brain. Data provided by the Psychoactive Drug Screening Program (PDSP).

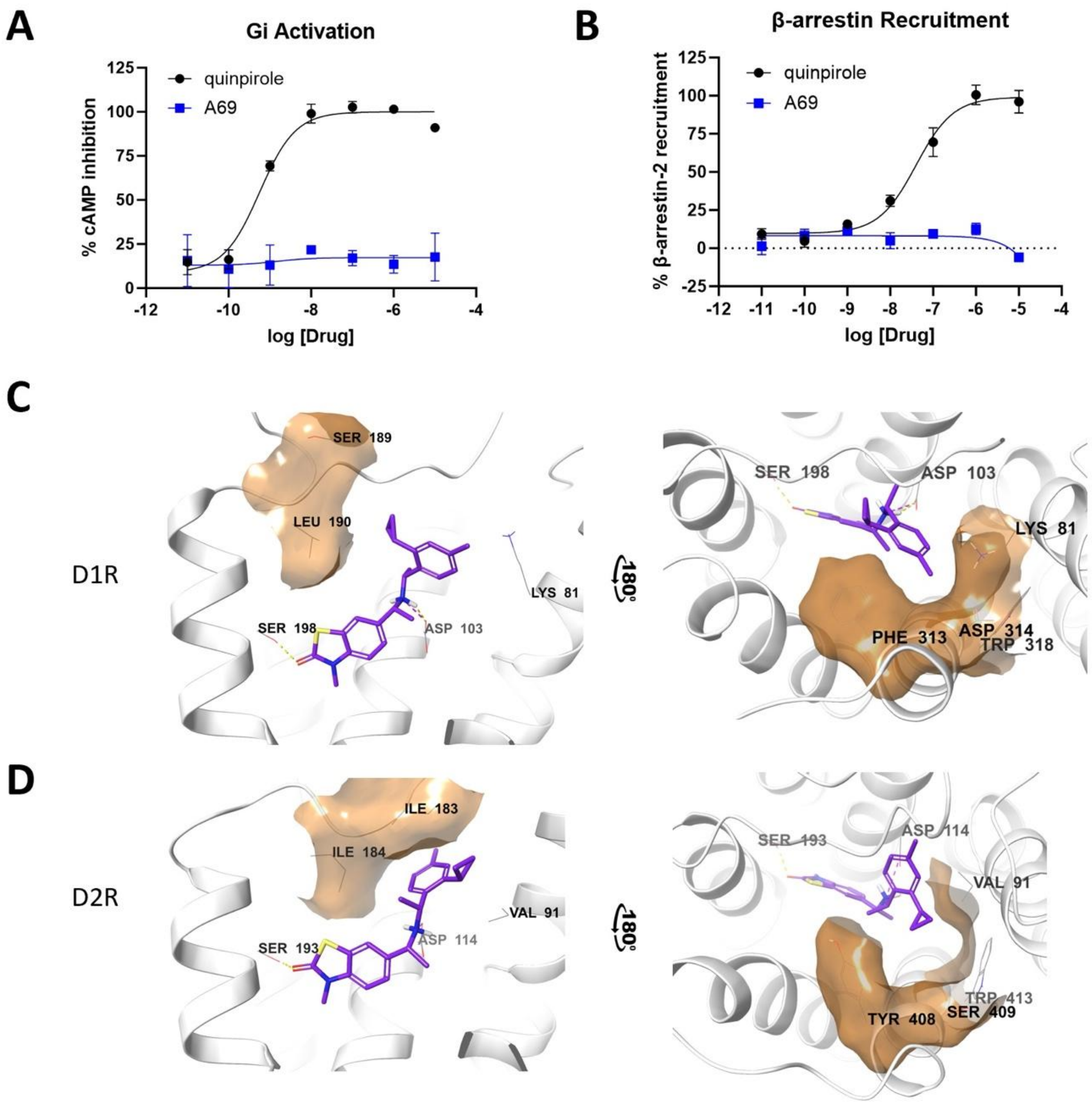

Supplementary Figure 6. Dose-response curves of A69 and quinpirole induced Gi activation (**A**) and β-arrestin recruitment (**B**) at D3R. (**C-D**) hD1R (PDB: 7JVQ) and hD2R structures (PDB: 8IRS). Docking of A69 at hD1R (C) and D2R (D). A69 is highlighted in purple, while the receptor surface area in the ECL2 region (left) or the EBP region (right) are in orange. N = 3 independent experiments. Error bars represent ± SD.

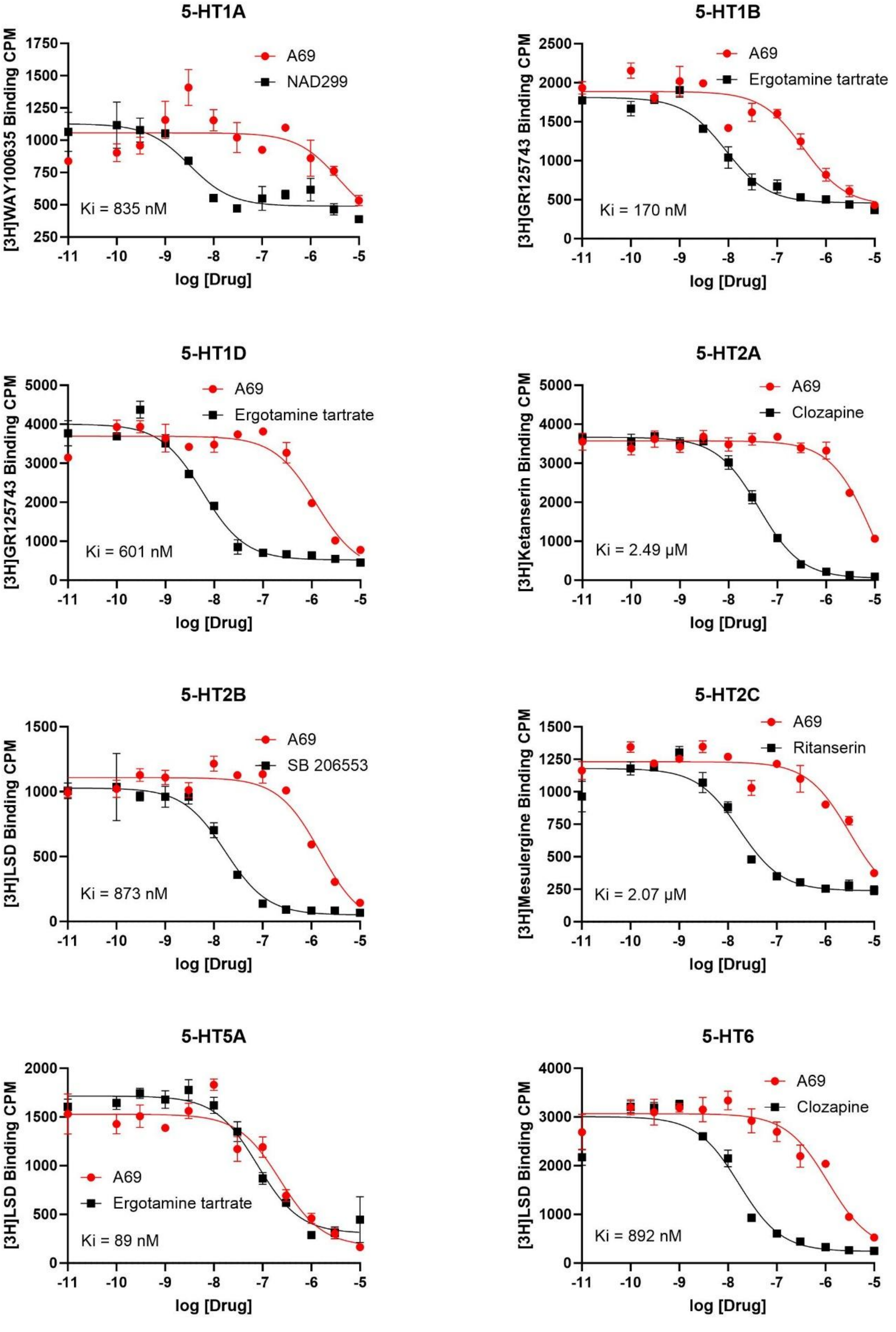
5-HT1A
[3H]WAY100635 Binding CPM
A69
NAD299
Ki = 835 nM
log [Drug]
5-HT1B
[3H]GR125743 Binding CPM
A69
Ergotamine tartrate
Ki = 170 nM
log [Drug]
5-HT1D
[3H]GR125743 Binding CPM
A69
Ergotamine tartrate
Ki = 601 nM
log [Drug]
5-HT2A
[3H]Ketanserin Binding CPM
A69
Clozapine
Ki = 2.49 μM
log [Drug]
5-HT2B
[3H]LSD Binding CPM
A69
SB 206553
Ki = 873 nM
log [Drug]
5-HT2C
[3H]Mesulergine Binding CPM
A69
Ritanserin
Ki = 2.07 μM
log [Drug]
5-HT5A
[3H]LSD Binding CPM
A69
Ergotamine tartrate
Ki = 89 nM
log [Drug]
5-HT6
[3H]LSD Binding CPM
A69
Clozapine
Ki = 892 nM
log [Drug]

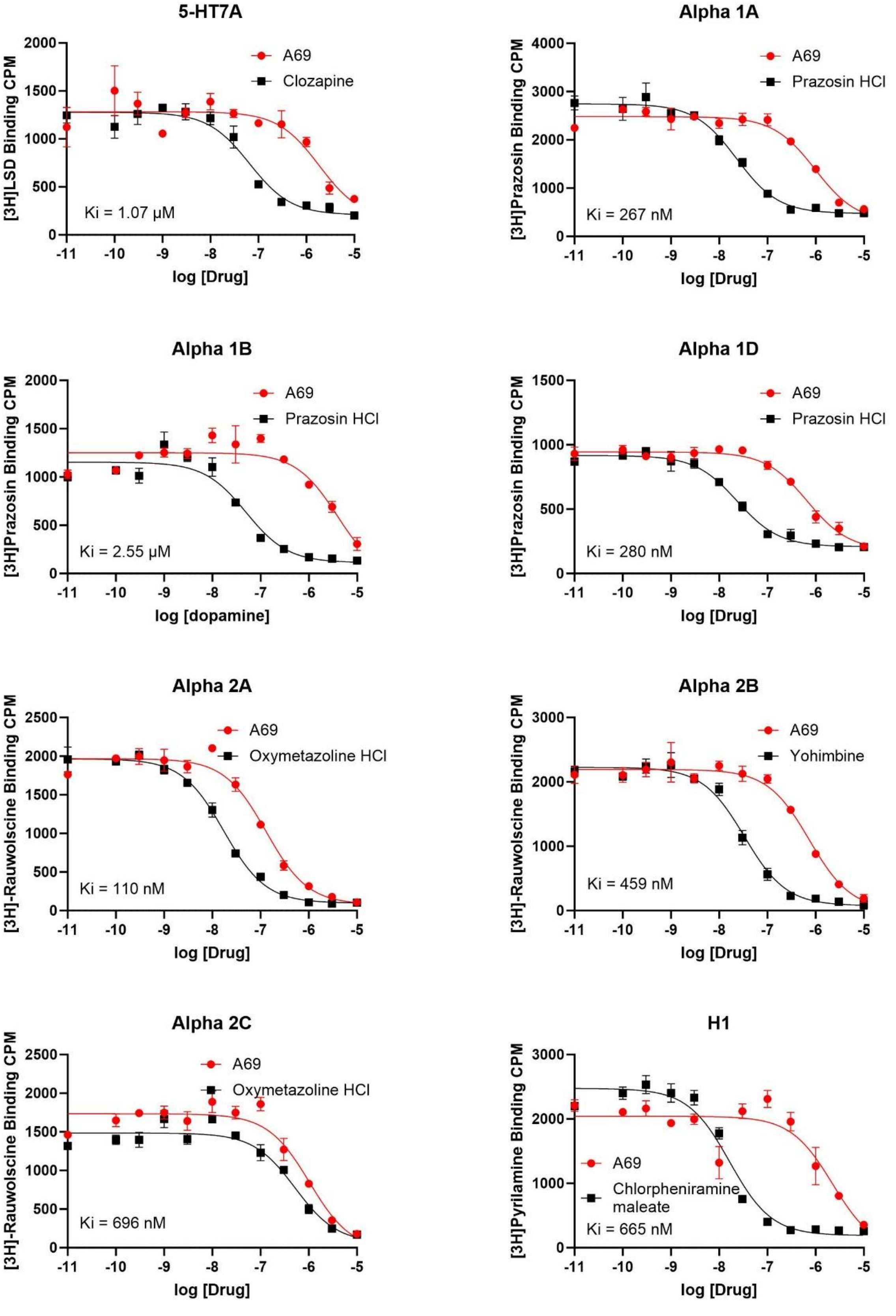
5-HT7A
[3H]LSD Binding CPM
A69
Clozapine
Ki = 1.07 μM
log [Drug]
Alpha 1A
[3H]Prazosin Binding CPM
A69
Prazosin HCl
Ki = 267 nM
log [Drug]
Alpha 1B
[3H]Prazosin Binding CPM
A69
Prazosin HCl
Ki = 2.55 μM
log [dopamine]
Alpha 1D
[3H]Prazosin Binding CPM
A69
Prazosin HCl
Ki = 280 nM
log [Drug]
Alpha 2A
[3H]-Rauwolscine Binding CPM
A69
Oxymetazoline HCl
Ki = 110 nM
log [Drug]
Alpha 2B
[3H]-Rauwolscine Binding CPM
A69
Yohimbine
Ki = 459 nM
log [Drug]
Alpha 2C
[3H]-Rauwolscine Binding CPM
A69
Oxymetazoline HCl
Ki = 696 nM
log [Drug]
H1
[3H]Pyrilamine Binding CPM
A69
Chlorpheniramine maleate
Ki = 665 nM
log [Drug]

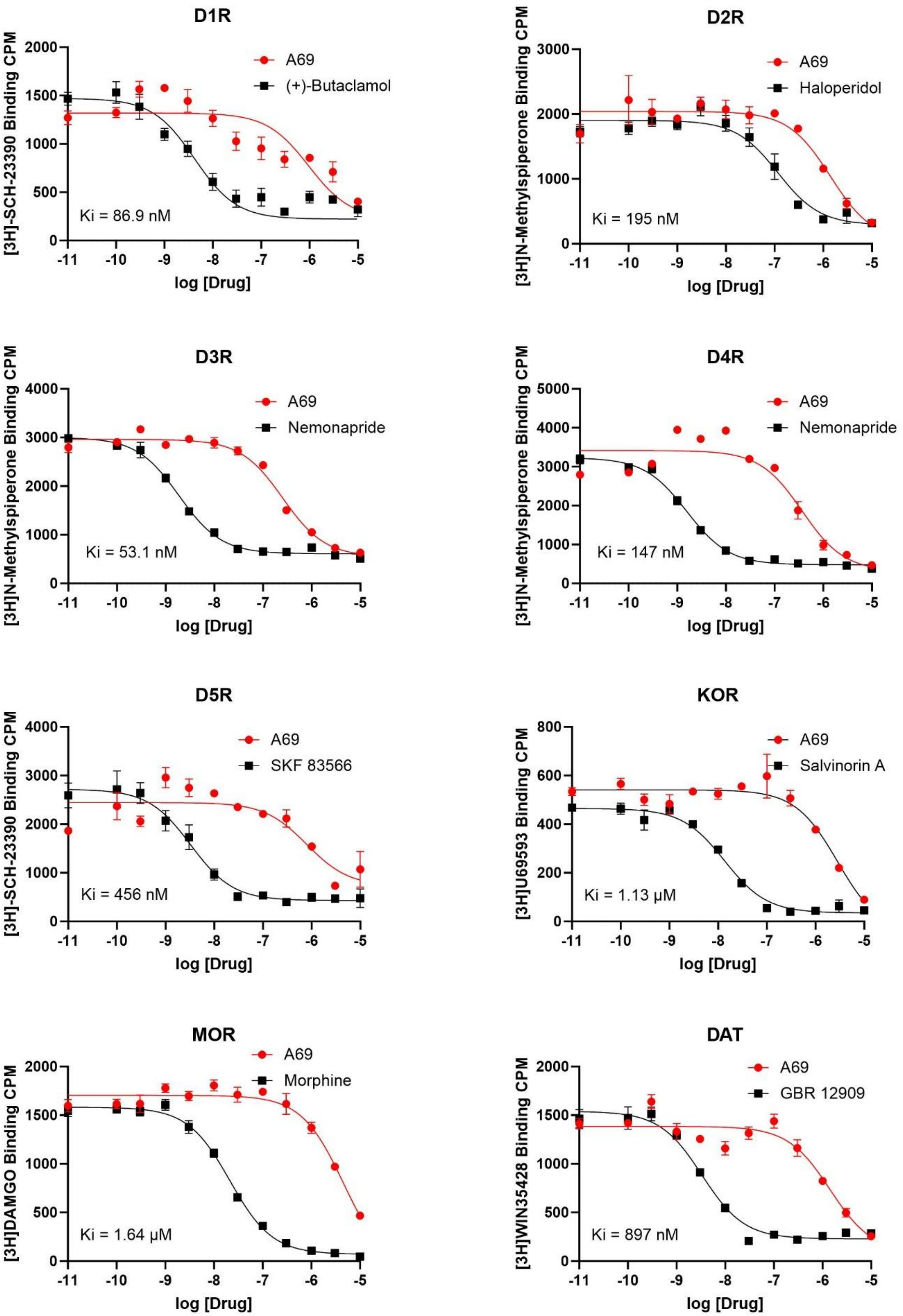
D1R
[3H]-SCH-23390 Binding CPM
A69
(+)-Butaclamol
Ki = 86.9 nM
log [Drug]
D2R
[3H]N-Methylspiperone Binding CPM
A69
Haloperidol
Ki = 195 nM
log [Drug]
D3R
[3H]N-Methylspiperone Binding CPM
A69
Nemonapride
Ki = 53.1 nM
log [Drug]
D4R
[3H]N-Methylspiperone Binding CPM
A69
Nemonapride
Ki = 147 nM
log [Drug]
D5R
[3H]-SCH-23390 Binding CPM
A69
SKF 83566
Ki = 456 nM
log [Drug]
KOR
[3H]U69593 Binding CPM
A69
Salvinorin A
Ki = 1.13 μM
log [Drug]
MOR
[3H]DAMGO Binding CPM
A69
Morphine
Ki = 1.64 μM
log [Drug]
DAT
[3H]WIN35428 Binding CPM
A69
GBR 12909
Ki = 897 nM
log [Drug]

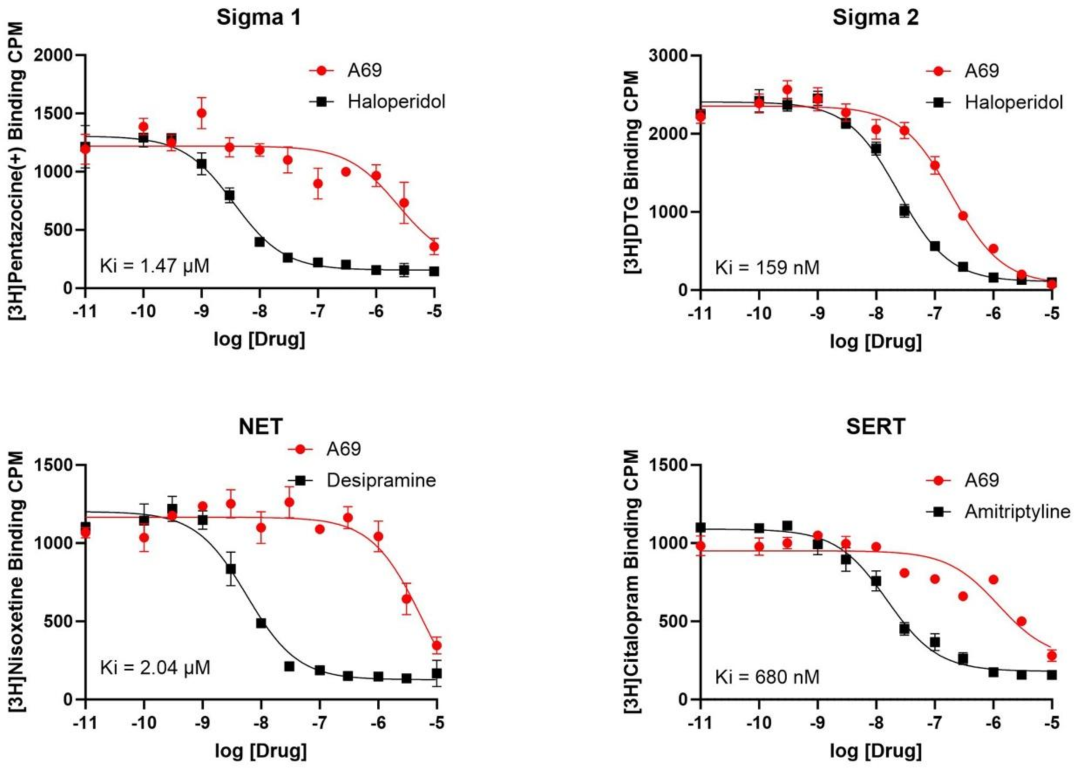

Supplementary Figure 7. Competitive radioligand binding assays testing dose-responses of A69 and control compounds at various receptor targets in the brain. Data provided by the Psychoactive Drug Screening Program (PDSP).

Supplementary Table 1. PDSP affinity screening of A82R at various receptor targets in the brain.

Receptor	logKi	Ki (nM)	Receptor	logKi	Ki (nM)	Receptor	logKi	Ki (nM)
SERT	-8.02	9.46	5-HT2C	> -5	> 10,000	D3R	> -5	> 10,000
Sigma 2	-7.05	88.7	5-HT3	> -5	> 10,000	D4R	> -5	> 10,000
KOR	-7.01	97.8	5-HT5A	> -5	> 10,000	D5R	> -5	> 10,000
Alpha2A	-6.26	544	5-HT6	> -5	> 10,000	H1	> -5	> 10,000
5-HT1B	-6.25	568	5-HT7A	> -5	> 10,000	H3	> -5	> 10,000
5-HT1A	-6.15	704	Alpha1A	> -5	> 10,000	H4	> -5	> 10,000
D1R	-6.13	733	Alpha1B	> -5	> 10,000	DOR	> -5	> 10,000
NET	-5.86	1381	Alpha1D	> -5	> 10,000	M1	> -5	> 10,000
Sigma 1	-5.51	3111	Alpha2B	> -5	> 10,000	M2	> -5	> 10,000
DAT	-5.34	4600	Beta1	> -5	> 10,000	M3	> -5	> 10,000
Alpha2C	-5.26	5489	Beta2	> -5	> 10,000	M4	> -5	> 10,000
5-HT1D	> -5	> 10,000	Beta3	> -5	> 10,000	M5	> -5	> 10,000
5-HT2A	> -5	> 10,000	BZP	> -5	> 10,000	MOR	> -5	> 10,000
5-HT2B	> -5	> 10,000	D2R	> -5	> 10,000	PBR	> -5	> 10,000

Supplementary Table 2. PDSP affinity screening of A69 at various receptor targets in the brain.

Receptor	logKi	Ki (nM)	Receptor	logKi	Ki (nM)	Receptor	logKi	Ki (nM)
D3R	-7.28	53.1	SERT	-6.17	680	5-HT3	>-5	>10000
D1R	-7.06	86.9	Alpha2C	-6.16	696	Beta1	>-5	>10000
5-HT5A	-7.05	88.8	5-HT1A	-6.08	835	Beta2	>-5	>10000
Alpha2A	-6.96	110	5-HT2B	-6.06	873	Beta3	>-5	>10000
D4R	-6.83	147	5-HT6	-6.05	892	BZP	>-5	>10000
Sigma 2	-6.8	159	DAT	-6.05	897	DOR	>-5	>10000
5-HT1B	-6.77	170	5-HT7A	-5.97	1067	H3	>-5	>10000
D2R	-6.71	195	KOR	-5.95	1134	H4	>-5	>10000
Alpha1A	-6.57	267	Sigma 1	-5.83	1467	M1	>-5	>10000
Alpha1D	-6.55	280	MOR	-5.79	1639	M2	>-5	>10000
D5R	-6.34	456	NET	-5.69	2045	M3	>-5	>10000
Alpha2B	-6.34	459	5-HT2C	-5.68	2065	M4	>-5	>10000
5-HT1D	-6.22	601	5-HT2A	-5.6	2488	M5	>-5	>10000
H1	-6.18	665	Alpha1B	-5.59	2553	PBR	>-5	>10000
